# Non-enzymatic conversion of RNA sequence information into DNA by squaramide ligation for accurate RNA quantification

**DOI:** 10.1038/s42004-026-02069-5

**Published:** 2026-05-19

**Authors:** Lapatrada Taemaitree, Friedrich Burba, Nina Harvey, Arun Shivalingam, Tom Brown, Afaf H. El-Sagheer

**Affiliations:** 1 Department of Chemistry, University of Oxford, Chemistry Research Laboratory, Oxford, UK; 2 Department of Integrated Science, Faculty of Science, Khon Kaen University, Khon Kaen, Thailand; 3 School of Chemistry and Chemical Engineering, University of Southampton, Highfield Campus, Southampton, UK; 4 Institute for Life Sciences, University of Southampton, Highfield Campus, Southampton, UK

**Keywords:** RNA, DNA, Chemical modification, Bioanalytical chemistry

## Abstract

RNA is an important biomarker for research and diagnostics. However, its transient nature and fragility require its conversion to DNA prior to detection. To this end, enzymatic approaches have been used but are limited by biological constraints, while chemical methods hold much promise but have not been competitive alternatives. Here, we demonstrate that chemical ligation of DNA oligonucleotides hybridised to a complementary RNA template to form an artificial squaramide backbone can be used to quantify sub-attomoles of RNA. This reaction requires mildly buffered, monovalent salt solutions with no extra chemical reagents, and can be performed at a range of temperatures within minutes. We describe the careful design of a three-component ligation system that minimises false-positives and demonstrate its use in qPCR to detect long RNAs in complex systems. Detection limits of 0.3 attomoles are achieved making it one of the most sensitive and specific chemical ligation systems to the best of our knowledge.

## Abstract (web summary)

Most quantitative RNA detection methods depend on enzymatic reverse transcription to convert RNA into DNA, but this process is subject to inherent biological limitations, and while chemical methods are promising to date none have been competitive alternatives. Here, the authors introduce a non-enzymatic chemical ligation strategy in which DNA oligonucleotides hybridised to a complementary RNA template form an artificial squaramide backbone, enabling highly sensitive RNA detection at sub-attomole levels comparable to enzymatic systems.

## Introduction

Our cells generate and destroy RNA molecules as their biological requirements change, while invasive pathogens leave traces of RNA as they proliferate. As a consequence, RNA levels are frequently monitored for research and diagnostic purposes. The majority of analytical techniques rely on an RNA-to-DNA conversion step before amplification, using methods such as qPCR, RPA, LAMP or SHERLOCK^1^. This conversion step is usually performed using reverse transcriptase enzymes. While effective and easy to use, these enzymes display biases that can mask differential expression of target genes, raise false positive conclusions and make inter-study comparisons challenging^2^. For example, an evaluation of eight commercially available reverse transcriptases demonstrated up to 100-fold variation in RNA quantification^3^, while comparison of various primer strategies for reverse transcription found that each strategy has a different impact on the expression of five different genes with variation as high as 24-fold^4^.

An alternative to reverse transcription is the direct hybridisation of a complementary oligonucleotide to the target RNA. However, there is a clear limitation to this approach in that oligonucleotide hybridisation is not always highly specific. To mitigate this issue, these techniques usually rely on stringent washing to remove non-specific interactions (e.g. microarrays^5^) or electrophoretically separating the desired target from the background (e.g. Northern blotting^6^, or use of DNA nanoconstructs^7^). These introduce extra experimental complexity, resulting in variable RNA quantification ( ~ 10^5^–10^8^ molecules; i.e. femto- to atto-mole). Furthermore, they do not reach the single molecule (yoctomole) gold standard of reverse transcription (RT) qPCR.

An interesting compromise between the two approaches is the use of oligonucleotide hybridisation to detect the target RNA, followed by ligation to generate a DNA template that is amplifiable by a DNA polymerase. To this end, enzymatic approaches have been explored with SplintR ligase enabling attomoles of short ~30-nt miRNAs^8,9^ or ~300-nt circRNA^10^ to be detected. While promising, the detection limit was strongly dependent on ligation temperature (18 to 37 °C) possibly due to RNA secondary structures inhibiting oligonucleotide hybridisation^9,10^. However, SplintR ligase loses activity at the high temperatures necessary to disrupt RNA secondary structures, and requires magnesium ions in the reaction, a supplement that strongly stabilises RNA secondary structures^11^. These enzymatic restrictions, coupled with the expense of ligases, means there is great scope for chemical alternatives.

Pure chemical ligation has been used to link oligonucleotides to form canonical or modified backbones. Of the wide range of reactions possible^12^, only a handful of linkages (amide^13^, phosphate^14^, phosphorothioate^15–17^, phosphoramidate^18–21^, triazole^22,23^ and urea^24^) can be read-through by polymerases. Of these linkages, only several can be prepared in situ without the need for further purification before amplification by polymerases^15–17,20,21^. Of these reactions, the reagents for the reaction have poor stability in solution^15,20^ or the reaction rate is very slow (hours)^16,17,21^. As a result, while chemical ligation reactions theoretically offer benefits over enzymatic RNA to DNA conversion, to date none have been practically used as even alternatives to enzymatic methods in sensitive, quantitative applications such as qPCR.

To this end, a promising chemical ligation system is squaramide formation. In its simplest form, this reaction requires two DNA oligonucleotides modified with commercially available monomers, one of which has a terminal 3'-amino-dT and the other a terminal 5'-amino-dT. Upon introduction of an RNA template, the high affinity of oligonucleotides for their complementary RNA target strongly favours hybridisation, which co-localises the amino groups for in situ reaction using squarate ester mediated ligation^24,25^. Alternatively, one of the two amino oligonucleotides can be pre-activated to form a stable mono-squaramide thereby removing the need for extra chemical reagents in the reaction, beyond the pH 8.5 buffer. The resulting artificial backbone is recognised by Vent (exo-) polymerase^24^. However, the limit of detection was only on the femtomole scale, far higher than the attomole scale of enzymatic ligation and yoctomole scale of enzymatic reverse transcription.

Herein, we demonstrate un-templated ligation limits the sensitivity of chemical ligation and explore strategies to overcome this hurdle. In doing so, we show chemical ligation is a feasible alternative to enzymatic methods in quantitative applications such as qPCR detection of RNA. Through careful design and optimisation, sub-attomoles of long ~2,500-nt RNAs are quantitatively detected in complex systems by dual squaramide ligation. The reaction requires only monovalent metal ions, it can be performed at a range of temperatures and durations, and can directly replace the reverse transcription step of RT-qPCR using a straightforward ‘mix and incubate’ protocol.

## Results and discussion

### Developing an optimal ligation system for RNA quantification

To design these ligation systems, each of the amino-oligonucleotides contains a ~ 20-nt region that can hybridize to the target RNA, and a further 10 nts that are not complementary to the RNA target. The goal of the 10 nts is to provide freedom in amplification primer design and reduce the constraints imposed by the target RNA. For example, for AT-rich target RNAs, these 10 nts can be GC-rich to achieve a target annealing temperature independent of the target RNA, while in the case of anticipated primer dimers, this region can be modified to minimise such products. More pertinent to this specific ligation system, these 10 nts allow primer redesign to minimise hybridisation of the two amino oligonucleotides to each other in the absence of template. Generally, we sought positive △G values at 40 °C when comparing hybridisation between the two oligonucleotides using *mfold* (http://www.unafold.org).  Finally, easy redesign of primers is useful when template contamination occurs in the laboratory, a not-uncommon scenario with no trivial solution for regular RT-qPCR systems beyond full-lab decontamination.

**Fig. 1:**
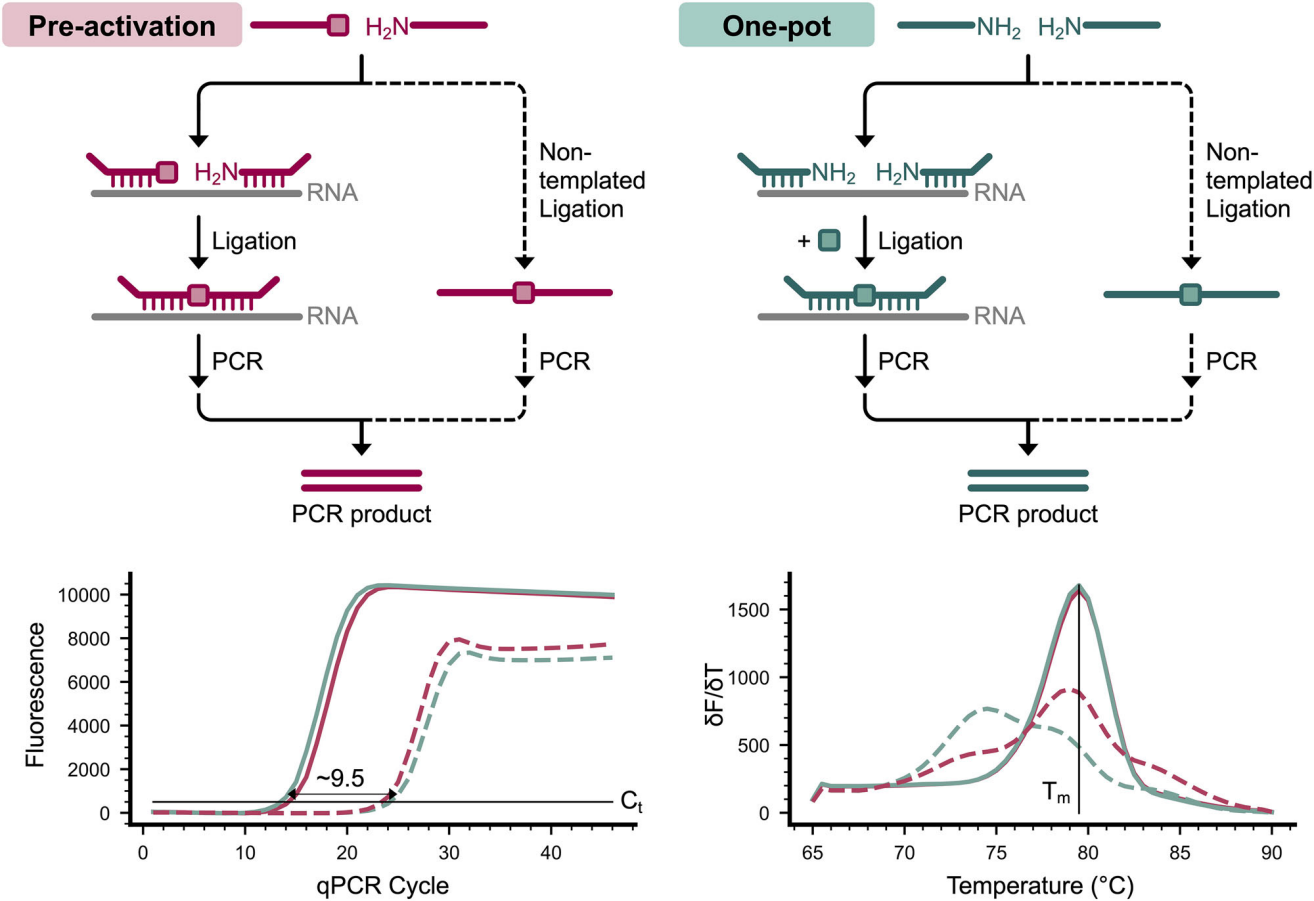
Single squaramide ligation performed using mono-squarate pre-activated oligonucleotides (pink) or with addition of squarate ester after RNA hybridisation in an in situ “one-pot” reaction (green). Neither mode of reaction displayed significant benefits in detection limits as assessed by the difference in the threshold signals of the template (solid lines) vs. no-template control (dashed lines) reactions. Ligations were performed at room temperature for 15 min and analysed by EvaGreen qPCR. The oligonucleotides used for ligation and qPCR as well as the target sequence can be found in Supplementary Table S1.

Based on these principles we synthesized oligonucleotides for squaramide ligation against a 2664-nt target RNA using standard methods (see Supplementary Methods; oligonucleotides and the target sequence are listed in Supplementary Table S1). To evaluate the design, we performed the ligation at room temperature for 15 min with 300 femtomoles of RNA. The reaction was halted by the addition of millimolar concentrations of ethanolamine, and analysed by qPCR using the intercalating dye EvaGreen. For optimisation purposes, we assumed that qPCR amplification and ligation efficiency are both quantitative. Therefore, comparison of the qPCR C_t_ value with and without the RNA target allows approximation of the limit of detection using the equation ${{RNA}}_{{in}}\times {2}^{{Ct}\left({RNA}\right)-{Ct}({NTC})}$^26^, where RNA_in_ is the initial quantity of RNA used in the template containing reaction. For the 3'-pre-activated mono-squaramide system (Fig. 1, pink), the hypothetical limit of detection was ~0.5 femtomoles, which is consistent with our previous results^24^. For the in situ system (Fig. 1, green), the hypothetical limit of detection was ~0.2 femtomoles. The melting curves of the PCR amplicons indicate that the no-template control amplicon had a similar melting temperature (T_m_) to that of the amplicon containing the RNA template in addition to non-specific product T_m_s. This suggested either the reactions were contaminated – unlikely given that the primer-only reactions did not show any amplification – or that untemplated ligation was a bottleneck to achieving lower detection limits.

**Fig. 2:**
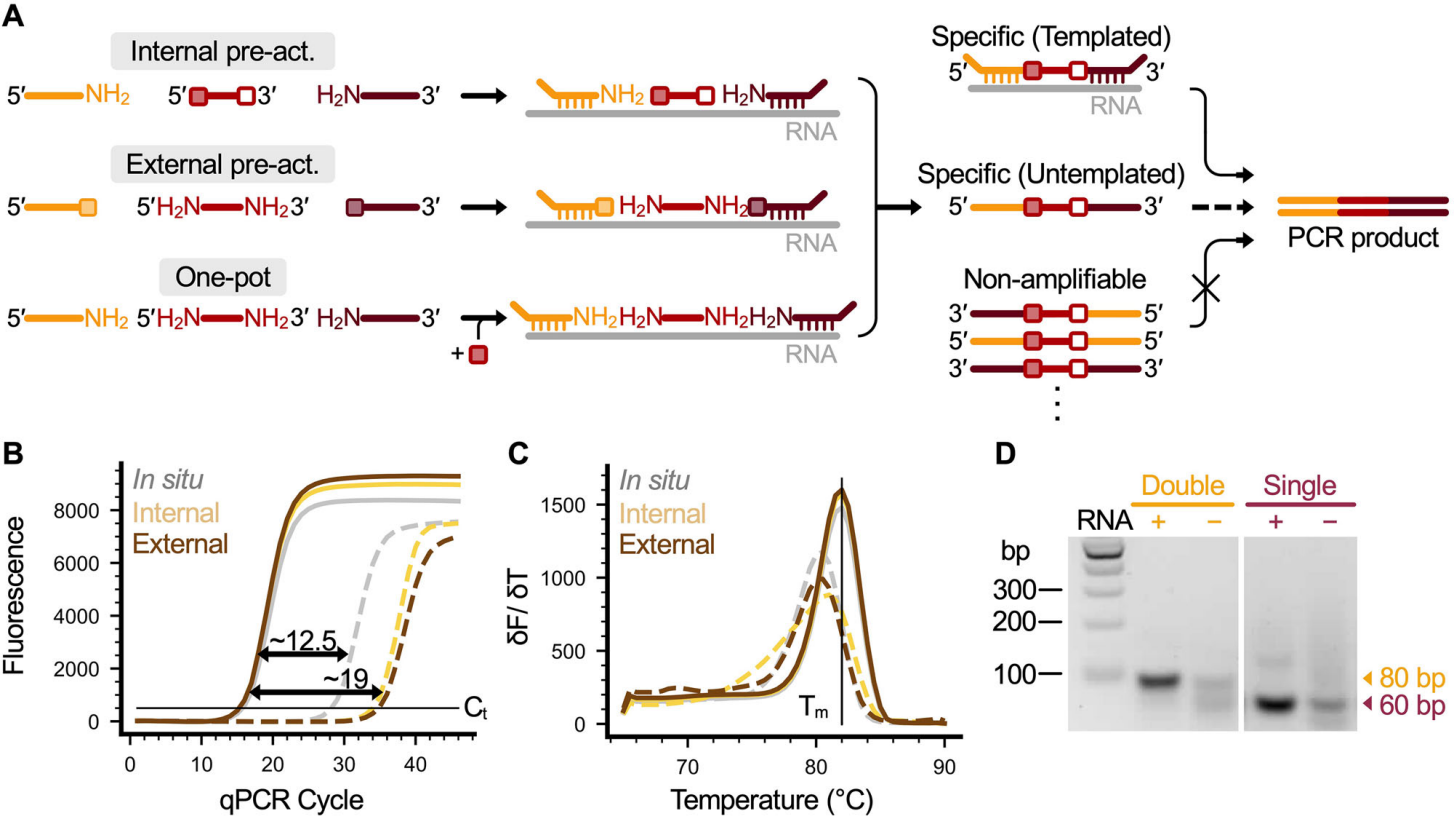
Dual squaramide ligation performed using mono-squarate pre-activated oligonucleotides (external = brown, internal = yellow) perform better than one-pot (grey) reactions for dual squaramide ligation. **A** Provides an overall schematic of the various ligation variations. **B** Demonstrates the theoretically wide detection range provided by pre-activated system (hypothetically 0.3 attomoles based on the equation ${{RNA}}_{{in}}\times {2}^{{Ct}\left({RNA}\right)-{Ct}({NTC})}$ where RNA_in_ is 300 femtomoles). **C** Untemplated reactions do not result in an amplicon of identical T_m_ to the expected product. **D** The significantly lower untemplated product formed by single (from Fig. 1) vs. dual ligation systems (internal) at the end point of qPCR by agarose gel electrophoresis. The oligonucleotides used for ligation and qPCR as well as the target sequence can be found in Supplementary Table S2.

To overcome the latter problem, we hypothesized that simultaneously performing two ligations instead of one and expanding the ligation system to include a ~ 20-nt central di-amino oligonucleotide between the two terminal mono-amino DNA oligonucleotides would reduce untemplated ligation; i.e. in order for a target to be recognised, three DNA components must be ligated together through two independent ligation reactions (Fig. 2A). Therefore, in the absence of a template, we expected trimolecular reaction kinetics with some reactions resulting in qPCR-incompetent templates due to incorrect 5'–3' directionality of the oligonucleotide components. On the other hand, in the presence of a template the reaction kinetics should remain pseudo-unimolecular due to the high affinity of oligonucleotides for their complementary strand and the rigid alignment and ordering of components it imposes (oligonucleotides and the target sequence are listed in Supplementary Table S2). The principle of multiple squaramide ligations against a single DNA template in a simple system has been reported^25^, but the reaction scope in terms of template dependence, template sensitivity and subsequent analysis for diagnostic purposes, for example, has not.

To evaluate this dual ligation system, we again compared C_t_ values for ligation reaction with and without a target RNA using qPCR. When ligation was performed in situ with squarate ester (Fig. 2B), the hypothetical limit of detection was improved from ~0.2 to ~0.05 femtomoles. Further optimisation of the squarate ester concentration in the in situ reaction (5 to 0.04 mM) failed to improve the hypothetical detection limit as the on-target ligation efficiency significantly dropped (Supplementary Fig. S1). However, the true strength of this design was revealed when the central di-amino oligonucleotide was pre-activated (Fig. 2B); hypothetical detection limits of ~0.0007 femtomoles or ~0.7 attomoles appeared feasible. Furthermore, minimal untemplated product was formed as the T_m_ for the no-template control is different to the product for the dual ligation system; cf. the single ligation system (Fig. 2C; supported by the gel in Fig. 2D). The pre-activation of the di-amino oligonucleotide was quantitative and there was no evidence of cyclisation of the oligonucleotide due to partial pre-activation of only one of the two amino groups as evaluated by UPLC-MS (Supplementary Fig. S2). The pre-activated di-mono-squaramide oligonucleotides were also stable during HPLC purification. Furthermore, there was no benefit to activating the two terminal amino oligonucleotides instead of just the central di-amino oligonucleotide, with hypothetical detection limits remaining at ~0.4 attomoles. Therefore, for convenience, all further studies used simple pre-activation of the central di-amino oligonucleotide in the dual ligation system.

### Verifying the robustness and sensitivity of the ligation system

**Fig. 3:**
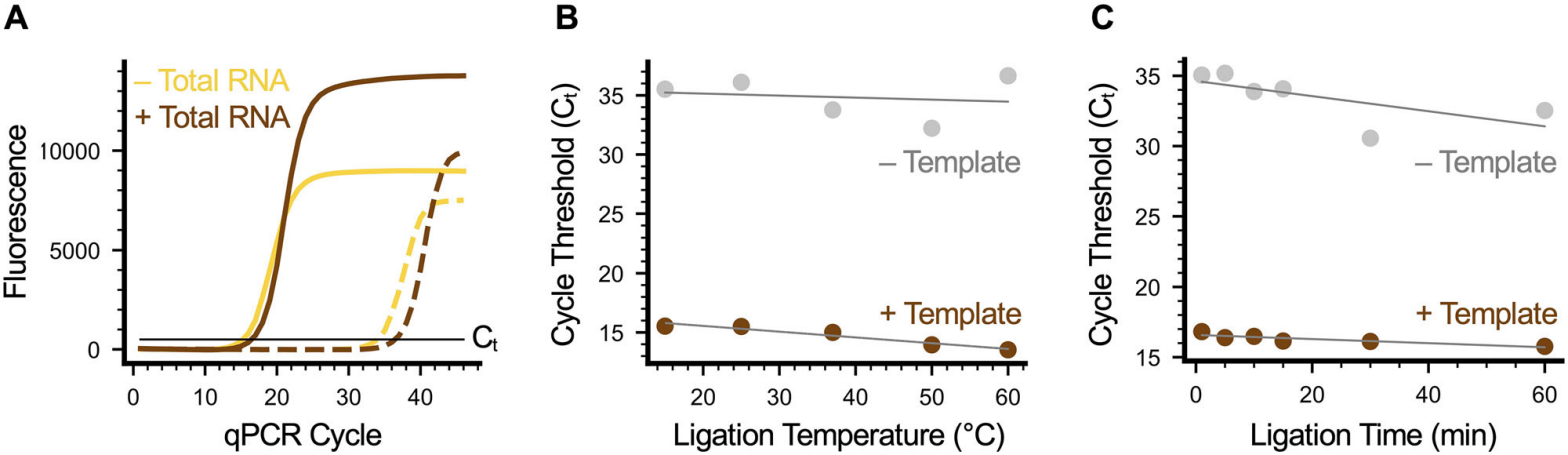
The negligible effect of background RNA, time and temperature on dual squaramide ligation. **A** Shows a small differences in C_t_ values for reactions with (brown) and without (yellow) 100 ng total RNA in the presence (solid lines) or absence (dashed lines) of target RNA. **B** Displays minimal temperature (15, 25, 37, 50, 60 °C for 15 min) dependence for squaramide ligation in the presence (brown spots, linear fit y = –0.049x + 16.52) or absence (grey spots, linear fit y = –0.017x + 35.49) of target RNA. **C** Demonstrates time (1, 5, 10, 15, 30 or 60 min at 50 °C) has a minimal impact on squaramide ligation in the presence (solid spots, linear fit y = –0.015x + 16.58) or absence (hollow spots, linear fit y = –0.054x + 34.63) of target RNA. The oligonucleotides used for ligation and qPCR as well as the target sequence can be found in Supplementary Table S2.

Next, we focused on the robustness of the assay. To demonstrate the specificity of ligation in a complex system, target RNA was spiked into 100 ng of total RNA extracted from MCF-7 cells (Fig. 3A). Pleasingly, the addition of total RNA to the assay, in the presence or absence of the target RNA, has no effect on the C_t_ value. This indicates that ligation is specific for the complementary RNA sequence. As a result, all subsequent reactions contained 100 ng of total RNA. An extra complexity in real systems is the secondary structure of the target RNA which may vary from target to target. Strongly structured RNAs could inhibit oligonucleotide hybridisation and therefore prevent ligation. To alleviate this problem, we investigated if it would be possible to perform ligation at higher temperatures (Fig. 3B). Promisingly, C_t_ values were relatively independent of ligation temperature (15, 25, 37, 50 or 60 °C for 15 min) in the presence and absence of the RNA template. As a consequence, all subsequent ligations were performed at 50 °C, a compromise between maintaining RNA stability and disrupting secondary structures. Finally, we evaluated the effect of reaction time (1, 5, 10, 15, 30 or 60 min at 50 °C, Fig. 3C). While it may be beneficial for the ligation to occur rapidly, practically it may be useful to perform the ligation for longer times to facilitate more complex assay setups. Encouragingly, reaction times had minimal effect on the C_t_ value for ligation in the presence or absence of the template. These experiments demonstrate that ligation can occur within 1 min, but the template levels are stable for up to 1 h in both template dependent and independent reaction. For all subsequent reactions, a reaction time of 15 min was chosen for convenience. The ability to optimise ligation efficiency by varying temperature over a wide range is a distinct advantage of chemical methods over enzymatic ligation.

**Fig. 4:**
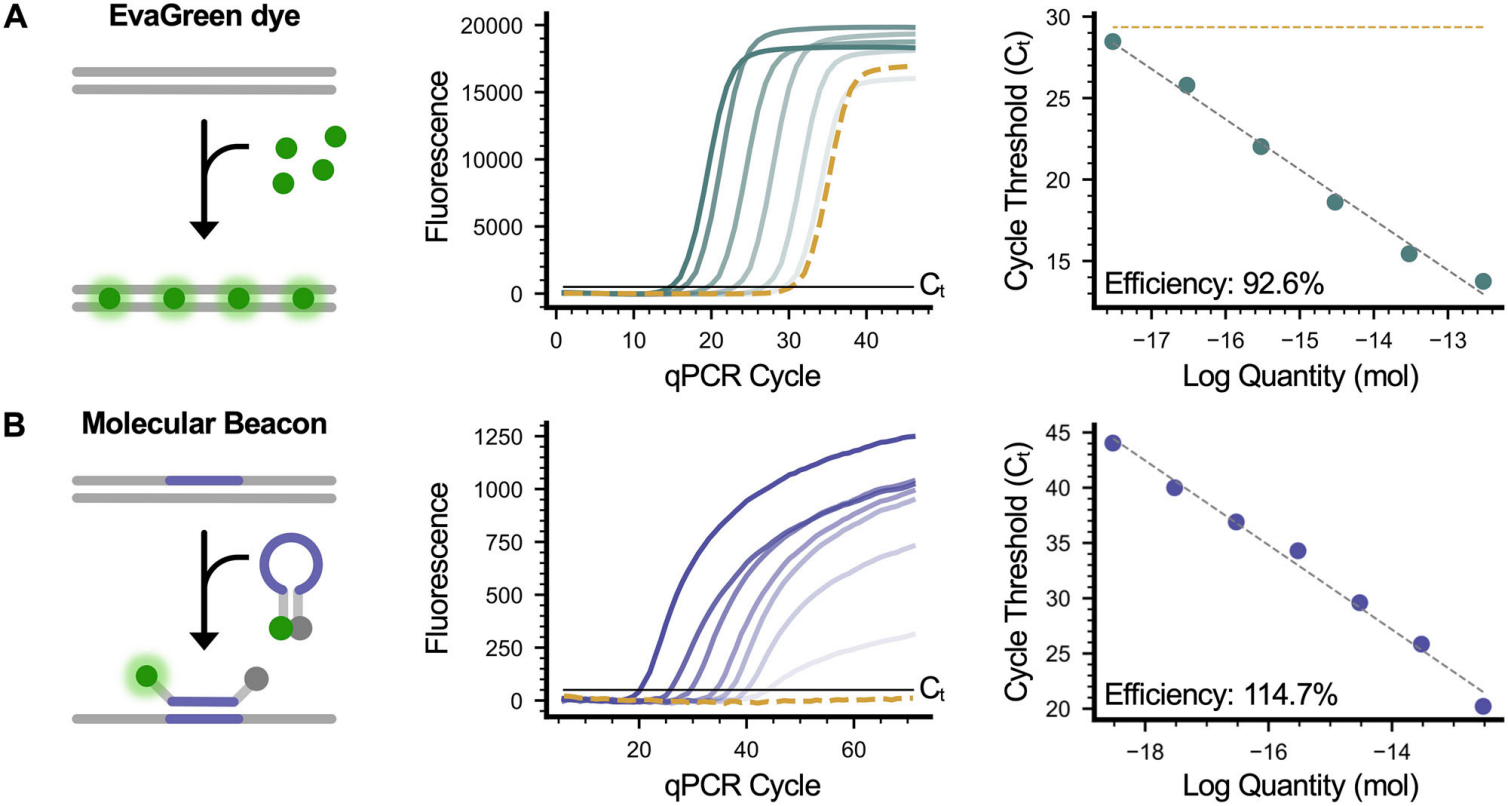
Quantitative detection of target RNA to DNA by squaramide ligation for mRNA site 1. Reactions were performed with target RNA (300, 30, 3, 0.3, 0.03, 0.003, 0.0003 femtomoles) in the presence of 100 ng total RNA from MCF-7 cells for 15 min at 50 °C. **A** qPCR reactions performed with EvaGreen intercalating dye (92.54% efficiency, R^2^ = 0.995) and **B** with Molecular Beacon (114.7% efficiency, R^2^ = 0.995). Yellow hashed line is the no-template negative control. The lack of amplification for the no-template Molecular Beacon reactions enabled one order of magnitude lower detection. More independent replicates can be found in Supplementary Fig. S3 (for EvaGreen; **A**) and Supplementary Fig. S6 (for Molecular Beacon; **B**). The oligonucleotides used for ligation against mRNA site 1 and qPCR as well as the target sequence can be found in Supplementary Table S2.

With the scope of the dual ligation system now explored, ligations were performed as a function of target RNA concentration to accurately determine the limits of detection. Each reaction had identical ligation conditions (0.3 µM oligonucleotide concentrations, 100 ng total RNA, 50 °C, 15 min), and the only variable was quantity of target RNA (300, 30, 3, 0.3, 0.03, 0.003 and 0.0003 femtomoles). Analysis of these reactions showed excellent qPCR efficiency (102.22 ± 7.13%, R^2^ = 0.991 ± 0.004, n (independent repeats) = 3, ± standard deviation; Fig. 4A for representative example; Supplementary Fig. S3) over six orders of magnitude of target RNA (300–0.003 femtomoles). This suggests that ligation is quantitative and that Vent (exo-) polymerase can read through the artificial backbone efficiently. To show the generality of the approach, a second set of oligonucleotides were prepared to target another RNA site. These results corroborated those of the first design showing almost identical efficiencies and limits of detection (93.21–99.24%, R^2^ = 0.993–0.999, n (independent repeats) = 2; Supplementary Fig. S4).

Unfortunately, for the lowest quantities (0.003 and 0.0003 femtomoles), the C_t_ values were too close to (within 3.3 cycles) or beyond the value of the no-template control. Interestingly, the T_m_ of the amplicons in the presence and absence of the template were different, suggesting non-specific – but not untemplated – products are limiting RNA detection. Non-specific products are common in qPCR at very high cycles ( > 30) and a simple solution is to use a probe that hybridises to the intended product rather than relying on non-specific intercalating dye such as EvaGreen.

### Refining the detection system to maximise sensitivity

For the squaramide ligation system, there is one limitation to bear in mind when deciding the type of probe to use: the artificial backbone is best tolerated (read-through) by Vent (exo-) polymerase^24^. This polymerase lacks 5' to 3' exonuclease activity and consequently cannot be used with TaqMan probes. However, Molecular Beacons^27^ – quenched hairpins that are opened in the presence of the target RNA – can be used. For the dual ligation system, this involved very little extra design as the central di-amino oligonucleotide must be present in any valid ligated product, and is approximately 20-nt long. Consequently, the non-stem hairpin sequence of the Molecular Beacon is complementary to that of the central di-amino oligonucleotide. We found optimisation of primer and Molecular Beacon concentrations was critical to successful qPCR amplification (Supplementary Fig. S5). Using the optimal qPCR conditions, we again held the ligation conditions constant (0.3 µM oligonucleotide concentrations, 100 ng total RNA, 50 °C, 15 min) and varied the target RNA quantity (300, 30, 3, 0.3, 0.03, 0.003, 0.0003 and 0.00003 femtomoles). Pleasingly the standard curves demonstrated excellent qPCR efficiency (110.01 ± 3.72%, R^2^ = 0.989 ± 0.005, n (independent repeats) = 3, ± standard deviation; Fig. 4B for representative example; Supplementary Fig. S6) including the previously obscured 0.003 and 0.0003 femtomole target RNA quantities. The background signal from the no-template control was almost negligible. Again, these results were reproducible against a second target site (101.02 ± 2.71%, R^2^ = 0.996 ± 0.001, n (independent repeats) = 3, ± standard deviation; Supplementary Fig. S7).

### Evaluating the fundamental chemistry of the ligation system

**Fig. 5:**
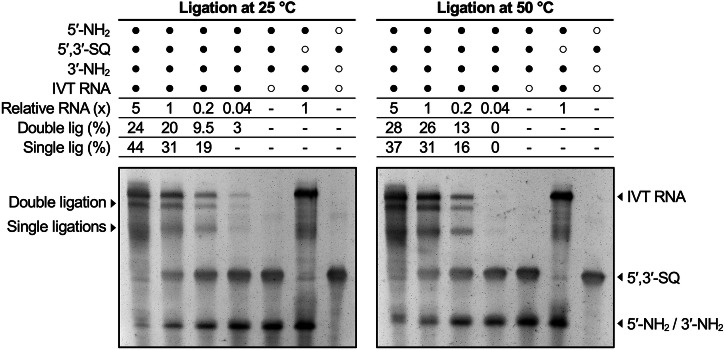
Dual squaramide ligation products were formed only in the presence of RNA targets at both room temperature (25 °C) and 50 °C. Reactions were performed with a 1:1:1 ratio 5',3'-di-squaramide (5',3'-SQ), 5’-amino (5'-NH_2_) and 3'-amino (3'-NH_2_) oligonucleotides (9 picomoles each) at 25 or 50 °C for 15 min. The relative amount of perfectly complementary target RNA to the oligonucleotides was varied from 45 to 0.36 picomoles (5, 1, 0.2 and 0.04 ×) to demonstrate rate-limiting cases. Reactions were analysed on 15% denaturing polyacrylamide gels post-stained with RedSafe dye. Black circles indicate a component was added to the reaction lane, while hollow white circles indicate the component was omitted. Conversion to the double and single ligation products was determined by dividing the intensity of the product band by the intensity of all other DNA oligonucleotide bands using ImageJ; note that samples derive from the same experiment and that gels were processed in parallel. Dashes indicate the value could not be determined. The oligonucleotides and templates for RNA generation can be found in Supplementary Table S4. The uncropped gels are in Supplementary Fig. S10 (A and B respectively).

It is interesting to note that we were unable to detect concentrations lower than 0.3 attomoles (0.0003 femtomoles), while on the other hand RT-qPCR could reliably detect 0.03 attomoles using the same Molecular Beacon detection chemistry (Supplementary Fig. S8) and in theory go to single molecule yoctomole limits. This suggests a fundamental limit has been reached for dual squaramide ligation. To investigate this and to corroborate the efficiency of the dual ligation system, the ligation products were directly analysed on denaturing polyacrylamide gels (Fig. 5 and Supplementary Fig. S9). For these studies, the primary goal was to clearly identify single vs. double vs. no ligation; therefore, oligonucleotides were redesigned to give a 77-nt double ligation product that is easily separable from 56/57-nt single ligation products that run as a single band. The secondary goal was to evaluate the specificity of ligation; therefore, shorter 86-nt RNA templates were prepared from in vitro transcription (IVT) of synthetic DNA containing different point mutations. Finally, ligations contained a 1:1:1 ratio of the three DNA oligonucleotides (9 picomoles each); therefore, the intensity of the product band divided by the intensity of all other DNA oligonucleotide bands gives the relative amount of a ligation product in the reaction.

As expected, the double ligation product was observed in all reactions when the amount of RNA template was also detectable (5, 1 and 0.2 × relative to the DNA oligonucleotides). At lower RNA template concentrations (0.04 ×), neither the RNA template nor the ligation products were clearly observed by gel staining and would require indirect amplification methods for accurate detection. On the other hand, the double ligation product was not the only product of the reaction; the amount of double ligation product was approximately equal to single ligation products at both room temperature and 50 °C for rate limiting RNA template concentrations (1 and 0.2 ×). This was unexpected and suggests templated ligation does not favour pseudo-unimolecular kinetics for the second ligation reaction. Previous studies have shown that artificial backbones generally destabilise duplex DNA^28,29^. As a consequence, we hypothesise that the first ligation either disrupts the hybridisation of the already hybridised strands to the template or that its formation interferes with the favourable orientation of the reactive groups for the second ligation reaction. In both cases, this would result in a second, more stringent rate-limiting ligation step. This perhaps explains the excellent specificity of dual ligation to its complementary strand (Supplementary Fig. S9); even a single internal C-U mismatch in the hybridisation region is energetic costly and prevents formation of the double ligation product. Yet this may also explain the lower limit of detection of 0.3 attomoles in qPCR; the extremely low template concentrations mean the formation of a stable complex for the second ligation may be inhibited. Therefore, to reduce the limit of detection further, new artificial backbone linkages or duplex-stabilising modifications such as LNA may be required.

Finally, we explored whether TaqMan probe assays could be performed by supplementing the reaction with Taq (exo + ) polymerase (0.025 U/µL; equal amount to Vent (exo-) polymerase). The hypothesis was that Vent (exo-) polymerase is required for the conversion of the squaramide containing template to the natural phosphodiester form, and that all subsequent amplification should be independent of the polymerase. Pleasingly, this was successful (Supplementary Fig. S11) and removed the need for asymmetric primer concentrations in the reaction while also simplifying the probe design to being a 20-nt sequence complementary to the central di-amino oligonucleotide (no extra stem loop bases were required unlike the Molecular Beacon probe).

## Conclusion

In summary, we have demonstrated squaramide ligation reactions can convert a target RNA sequence to DNA in a complex mixture of RNAs for quantitative PCR detection. However, un-templated ligation limits the sensitivity of method and results in false positives at very low target RNA concentrations. To overcome this limitation, we developed a dual ligation system, which requires two independent chemical ligation reactions to happen to generate DNA products that is amplifiable. In the absence of a template RNA, this reaction is extremely unfavourable and effectively removes the false positives when coupled with probes for specific amplicon detection. This strategy is in principle extendable to other chemical ligation systems.

For squaramide ligation in particular, the reaction is simple to setup and requires no post-reaction clean up. It occurs within minutes and is insensitive to reaction time (up to 60 min) and relatively insensitive to reaction temperature (up to 60 °C). The resulting DNA contains an artificial backbone that is tolerated by DNA polymerases allowing standard qPCR assays to be performed. In conjunction with Molecular Beacons, the quantitative detection limit was 0.3 attomoles, making it one of the most sensitive known chemical ligation systems.

## Methods

### Amino oligonucleotide pre-activation

3,4-Dimethoxy-3-cyclobutene-1,2-dione (100 mM in water made fresh, 2.5 µL) was added to the amino-modified DNA oligonucleotide (5000 pmol, 44.5 µL) in sodium borate buffer (0.5 M, pH 8.5, 3 µL). After incubation for 2 h at room temperature, samples were diluted with water and desalted using a NAP-10 column (G.E. Healthcare Life Sciences, GE17-0854-02) to yield the mono-squaramide oligonucleotide. Further purification by RP-HPLC was performed to ensure removal of residual 3,4-dimethoxy-3-cyclobutene-1,2-dione as described in the Supplementary Methods*Oligonucleotide purification* section. The purified mono-squaramide oligonucleotide could be freeze-dried or frozen without loss in activity.

### Pre-activated single ligation system

Ligation reactions consisted of 3'-pre-activated mono-squaramide oligonucleotide (0.3 µM, 1 µL), 5'-amino oligonucleotide (0.3 µM, 1 µL), 2,664-mer IVT RNA sample (300 or 0 femtomoles), sodium borate buffer (0.5 M, pH 8.5, 0.3 µL), sodium chloride (4 M, 0.5 µL) and water (up to 10 µL). The pre-activated mono-squaramide oligonucleotide was added to the reaction last, before incubation at room temperature for 15 min to allow hybridisation and spontaneous ligation. Reactions were quenched by the addition of ethanolamine (1 M, pH 8.5, 1 µL, 15 min, room temperature). The ligation mixture was then diluted 100-fold before qPCR analysis.

### In situ “one-pot” single ligation system for qPCR

5'-amino oligonucleotide (0.3 µM, 1 µL), 3'-amino oligonucleotide (0.3 µM, 1 µL), IVT RNA sample (300 or 0 femtomoles), sodium borate buffer (0.5 M, pH 8.5, 0.3 µL), sodium chloride (4 M, 0.5 µL) and water (up to 9 µL) were mixed and incubated for 15 min at room temperature. Next, 3,4-dimethoxy-3-cyclobutene-1,2-dione (50, 2, 0.4 mM, 1 µL) was added before further incubation for 15 min at room temperature to allow ligation. Reactions were quenched by the addition of ethanolamine (1 M, pH 8.5, 1 µL, 15 min, room temperature). The ligation mixture was then diluted 100-fold prior to qPCR.

### Pre-activated dual ligation system for qPCR

Ligation reactions consisted of 5'-3'-di-amino oligonucleotide (0.3 µM, 1 µL), 5'-amino oligonucleotide (0.3 µM, 1 µL), 3'-amino oligonucleotide (0.3 µM, 1 µL), IVT RNA sample (300, 30, 3, 0.3, 0.03, 0.003, 0.0003 or 0 femtomoles), sodium borate buffer (0.5 M, pH 8.5, 0.3 µL), sodium chloride (4 M, 0.5 µL), total RNA (if applicable, 100 ng/µL, 1 µL), Murine RNase inhibitor (only when total RNA was used, 4 U/µL, 1 µL, NEB, cat. no. M0314S) and water (up to 10 µL). Note that pre-activated mono-squaramide or di-squaramide oligonucleotides were used as appropriate and added as the final component to the reaction after the mixture was pre-incubated at the appropriate temperature (15, 25, 37, 50 or 60 °C). Reactions were then allowed to proceed for the desired amount of time (1, 5, 10, 15, 30 or 60 mins) at this temperature before being quenched by the addition of ethanolamine (1 M, pH 8.5, 1 µL, 15 min, room temperature). The ligation mixture was then diluted 100-fold before qPCR analysis.

### Pre-activated dual ligation system for denaturing PAGE

Ligation reactions consisted of 5'-3'-di-squaramide oligonucleotide (9 µM, 1 µL), 5’-amino oligonucleotide (9 µM, 1 µL), 3’-amino oligonucleotide (9 µM, 1 µL), IVT RNA (15, 3, 0.6 or 0.12 µM, 3 µL), sodium borate buffer (0.5 M, pH 8.5, 0.3 µL), sodium chloride (4 M, 0.5 µL) and water (up to 10 µL). Note that pre-activated di-squaramide oligonucleotide was added as the final component to the reaction after the mixture was pre-incubated at the appropriate temperature (25 or 50 °C). Reactions were then allowed to proceed for 15 min at this temperature before being quenched by the addition of ethanolamine (1 M, pH 8.5, 1 µL, 15 min) followed by an equal volume of formamide (11 µL). Entire reactions were loaded on 15% denaturing polyacrylamide gels and run at 5 W until the loading dye reached the end of the gel. Gels were then post-stained with 1× Vivantis RedSafe nucleic acid stain and visualised using a SynGene Gel Documentation system. Data were analysed as detailed in the Supplementary Methods*Denaturing PAGE analysis of ligation products* section.

### One-pot dual ligation system for qPCR

Similar to the one-pot single ligation system, 5'-amino oligonucleotide (0.3 µM, 1 µL), 5'-3'-di-amino oligonucleotide (0.3 µM, 1 µL), 3'-amino oligonucleotide (0.3 µM, 1 µL), IVT RNA sample (300 or 0 femtomoles), sodium borate buffer (0.5 M, pH 8.5, 0.3 µL), sodium chloride (4 M, 0.5 µL), total RNA (if applicable, 100 ng/µL, 1 µL), Murine RNase inhibitor (only when total RNA was used, 4 U/µL, 1 µL, NEB, cat. no. M0314S) and water (up to 9 µL) were mixed and incubated for 15 min either at room temperature. Next, 3,4-dimethoxy-3-cyclobutene-1,2-dione (100 mM in water made fresh, 1 µL) was added before further incubation for 15 min at room temperature. Samples were quenched by adding ethanolamine (1 M, pH 8.5, 1 µL) and incubated for 15 min at room temperature. The ligation mixture was diluted 100-fold prior to qPCR.

### Reporting summary

Further information on research design is available in the Nature Portfolio Reporting Summary linked to this article.

## Supplementary information

Transparent Peer Review file

Supplementary Information

Description of Additional Supplementary Files

Supplementary Data 1

Reporting summary

## Data Availability

All raw numerical data for graphs are included in Supplementary Data 1 file, while uncropped raw gels are in the Supplementary Information S10.
