# Supplementary material for: Non-enzymatic conversion of RNA sequence information into DNA by squaramide ligation for accurate RNA quantification
Source: Commun Chem. 2026 May 19;9:272. doi: 10.1038/s42004-026-02069-5 (PMC13454357; doi:10.1038/s42004-026-02069-5)
Supplement: Supplementary file 1 [file 42004_2026_2069_MOESM1_ESM.pdf]

nature portfolio

Peer Review File

Non-enzymatic conversion of RNA sequence information into DNA by squaramide ligation for accurate RNA quantification

Corresponding Author: Professor Afaf El-Sagheer

This file contains all reviewer reports in order by version, followed by all author rebuttals in order by version.

Version 0:

Reviewer comments:

Reviewer #1

(Remarks to the Author)
The manuscript by Taemaitree et al. reports RNA quantification technique using squaramide-ligation chemistry for RNA to DNA conversion, followed by qPCR. DNA fragments for chemical ligation were designed to complementary to target RNA, which realizes target RNA-directed reaction. The authors first attempted RNA detection using the previously reported methods: pre-activated ligation system and in situ “One-Pot” ligation system. The authors revealed that the detection limits of these strategies were sub-femtomole order. However, non-specific amplification was observed in the absence of a template. To solve this problem, the authors designed dual ligation system in which three fragments were ligated on the target RNA. This new strategy successfully achieved sub-attomoles detection of target RNA.
This novel strategy promises effective RNA detection, and the manuscript is well-written. However, some additional experiments and revisions are required before the publication in Communications Chemistry.

- Mismatch discrimination
One of the key factors in RNA detection is mismatch recognition. Ligase reaction is highly sensitive to mismatch: as an example, ligase-based technique shown in ref12 (RNA, 2019) demonstrated discrimination of Let 7 family with only partial mismatches. In contrast, the chemical strategy, including squaramide-ligation in the manuscript by the authors, is likely far less sensitive to mismatches. If this is not the case, the authors should present results demonstrating 1–3 mismatches in target RNA. If the authors cannot show results indicating mismatch recognition equivalent to or better than ligase, this possibility of low mismatch recognition should be discussed in the manuscript.

- Ligation efficiency
The authors primarily evaluated the reaction using Ct values. However, the current strategy relies mainly on ligation efficiency, which was not evaluated throughout the paper. The authors should provide the PAGE data after ligation to evaluate ligation efficiency, as shown in the previous paper (ref 23, Angew. Chem. Int. Ed. 2020). Such basic experiments are very important to understand the results and establishment of convincing methodology.

Minor points:
-In Fig. 2A, the NH_2 group on the first yellow chain is missing.
-In Fig. 3c, the x-axis should represent time, not temperature.

Reviewer #2

(Remarks to the Author)
The manuscript by Taemaitree and colleagues reports an RNA qPCR strategy using chemical ligation instead of reverse transcription. In my opinion the study fits in the area of analytical chemistry instead of biochemistry, because the reaction has been reported by the authors and others before. My comments are all based on this logic.

The authors accused reverse transcriptase for being biased during introduction. Though this is true, part of the problem roots from the choice of reagent or strategy. This is intrinsic for every kind of assay, including the study being reviewed here. The true problem is the bias among different genes, which is not covered in this study. The manuscript lacks direct comparison

with enzymatic reaction (reversed transcription or ligase). Therefore, within the scope of this manuscript, chemical ligation demonstrated no advantage over reverse transcription regarding “bias” or any other metrics.

The claim of limit of detection by the authors is not convincing. The calculation using no-template sample Ct value is problematic, particularly for EvaGreen qPCR. In Figure 2B, no-template sample Ct values were way over 30, making the values not reliable. Very high Ct values suggest a few copies before qPCR, making the amplification success rate during the first few cycles Boolean. Figure 3B and 3C are good examples. Furthermore, the no-template samples gave actual Ct values. In Figure 4A, the signal of 0.003 fmoles sample was very close to the no-template sample. Imagine a real sample giving out such a curve, it would be difficult to claim a positive detection. Figure 4B using molecular beacon presented much reliable demonstration of limit of detection. Nevertheless, typical RT-qPCR achieves much lower limit of detection (a few dozens of molecules), out-performing the strategy reported here.

The problem of Figure 4B is the assay stability. The Ct values of 10-fold diluted samples were not uniformly distributed. The instability may root from low ligation efficiency. Given the number of input molecules, the qPCR Ct values should be much lower than what Figure 4A shows, even considering the 100-fold dilution. For example, 0.003 fmoles after 100-fold dilution should be roughly 1E4 molecules, not concordant with Ct value of 30.

In summary, the authors reported an RNA qPCR strategy using chemical ligation. The strategy works, but demonstrating no advantage over current strategies.

Version 1:

Reviewer comments:

Reviewer #1

(Remarks to the Author)
The authors have responded thoughtfully to all of the reviewers' comments and presented the results of appropriate additional experiments. These results have been deeply discussed, and the manuscript has now been significantly improved. I totally agree to accept the current manuscript for the publication in Communications Chemistry.

Reviewer #2

(Remarks to the Author)
The authors have addressed my concerns.

Open Access This Peer Review File is licensed under a Creative Commons Attribution 4.0 International License, which permits use, sharing, adaptation, distribution and reproduction in any medium or format, as long as you give appropriate credit to the original author(s) and the source, provide a link to the Creative Commons license, and indicate if changes were made.
In cases where reviewers are anonymous, credit should be given to 'Anonymous Referee' and the source.

The images or other third party material in this Peer Review File are included in the article's Creative Commons license, unless indicated otherwise in a credit line to the material. If material is not included in the article's Creative Commons license and your intended use is not permitted by statutory regulation or exceeds the permitted use, you will need to obtain permission directly from the copyright holder.
To view a copy of this license, visit https://creativecommons.org/licenses/by/4.0/

Dear Dr Guo,

Re: Manuscript COMMSCHEM-25-1192

We would like to thank you and the reviewers for the time and effort put into reviewing our manuscript. We appreciate the insightful feedback, which helped us to strengthen and improve the manuscript. The additions we have made in the manuscript are highlighted in yellow.

Please find below our detailed point-by-point response to the reviewers' comments.

Reviewer 1.

1. **Mismatch discrimination**. One of the key factors in RNA detection is mismatch recognition. Ligase reaction is highly sensitive to mismatch [...] In contrast, the chemical strategy, including squaramide-ligation in the manuscript by the authors, is likely far less sensitive to mismatches. If this is not the case, the authors should present results demonstrating 1–3 mismatches in target RNA. If the authors cannot show results indicating mismatch recognition equivalent to or better than ligase, this possibility of low mismatch recognition should be discussed in the manuscript.

Our response to this point is combined with point 2 below.

2. **Ligation efficiency**. The authors primarily evaluated the reaction using C_t values. However, the current strategy relies mainly on ligation efficiency, which was not evaluated throughout the paper. The authors should provide the PAGE data after ligation to evaluate ligation efficiency, as shown in the previous paper (ref 23, Angew. Chem. Int. Ed. 2020). Such basic experiments are very important to understand the results and establishment of convincing methodology.

We thank the reviewer for suggesting these fundamental experiments (points 1 and 2), which we have performed and support our existing results. We have directly evaluated the squaramide ligation efficiency as a function of:

1. Reaction temperature (25 and 50 °C)
2. RNA target concentration (5, 1, 0.2 and 0.04 X relative to the ligation oligonucleotides)
3. Mismatches in the RNA target (0, 1, 2 or 3)

Briefly, these results demonstrate:

1. The second ligation reaction is less efficient than the first, adding an extra level of stringency to RNA detection that we had not originally anticipated
2. This stringency means even a single point mutation prevents formation of the dual ligation product

Our more detailed results and their interpretation are now included as follows (methods omitted for brevity):

> To corroborate the efficiency of the dual ligation system, the ligation products were directly analysed on denaturing polyacrylamide gels (Fig. 5 and Fig. S9). For these studies, the primary goal was to clearly identify single *vs.* double *vs.* no ligation; therefore, oligonucleotides were redesigned to give a 77-nt double ligation product that is easily separable from 56/57-nt single ligation products that run as a single band. The secondary goal was to evaluate the specificity of ligation; therefore, shorter 86-nt RNA templates were prepared from IVT of synthetic DNA containing different point mutations. Finally, ligations contained a 1:1:1 ratio of the three DNA oligonucleotides (9 picomoles each); therefore, the intensity of the product band divided by the

intensity of all other DNA oligonucleotide bands gives the relative amount of a ligation product in the reaction.

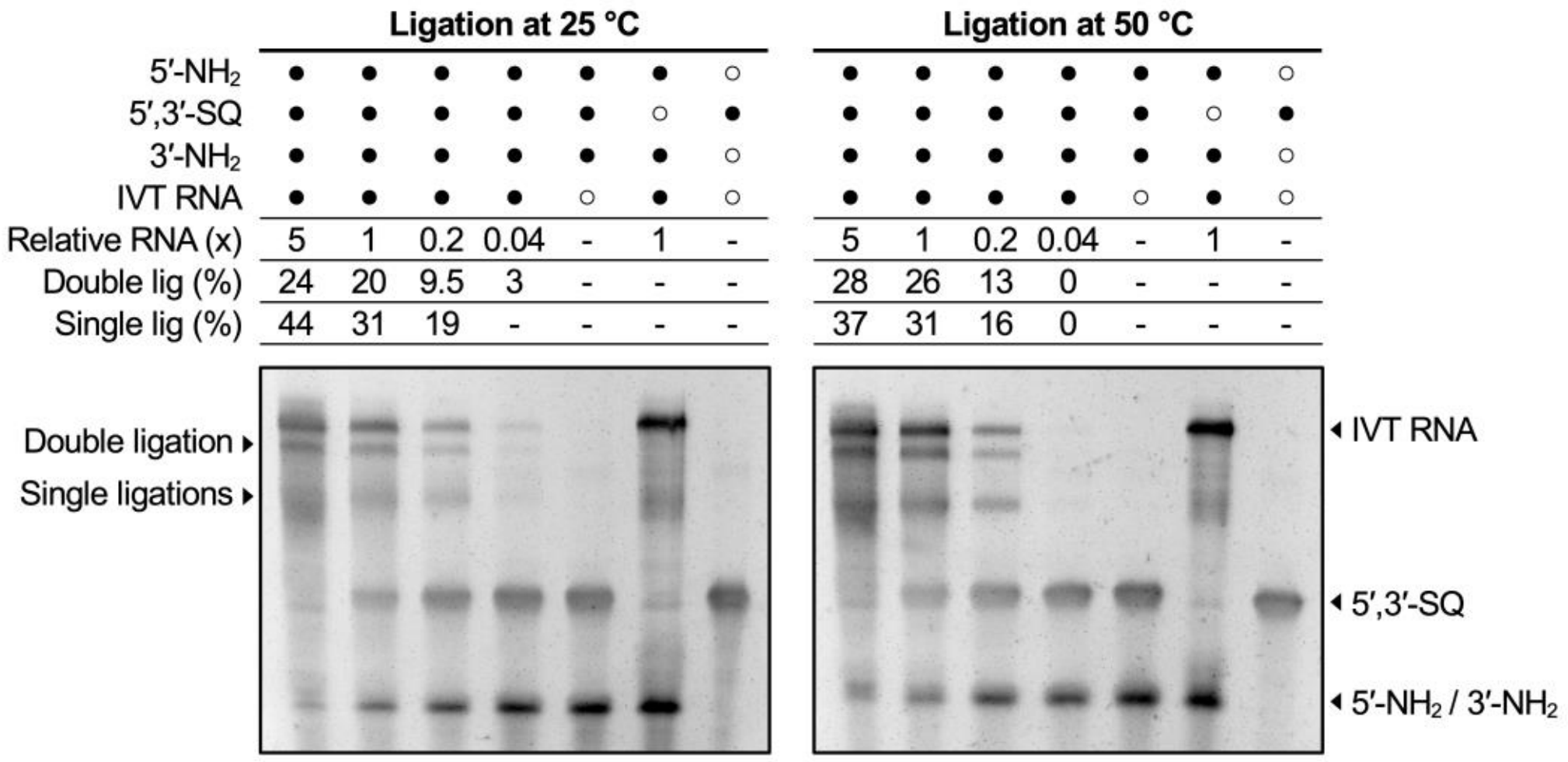

Figure 5. Dual squaramide ligation products were formed only in the presence of RNA targets at both room temperature (25 °C) and 50 °C. Reactions were performed with a 1:1:1 ratio 5',3'-di-squaramide (5',3'-SQ), 5'-amino (5'-NH_2), 3'-amino (3'-NH_2) oligonucleotides (9 picomoles each) at 25 or 50 °C for 15 min. The relative amount of perfectly complementary target RNA to the oligonucleotides was varied from 45 to 0.36 picomoles (5, 1, 0.2 and 0.04 X) to demonstrate rate-limiting cases. Reactions were analysed on 15 % denaturing polyacrylamide gels post-stained with RedSafe dye. Black circles indicate a component was added to the reaction lane, while hollow white circles indicate the component was omitted. Conversion to the double and single ligation products was determined by dividing the intensity of the product band by the intensity of all other DNA oligonucleotide bands using ImageJ. Dashes indicate the value could not be determined. The oligonucleotides and templates for RNA generation can be found in Supplementary Table S4.

As expected, the double ligation product was observed in all reactions when the amount of RNA template was also detectable (5, 1 and 0.2 X relative to the DNA oligonucleotides). At lower RNA template concentrations (0.04 X), neither the RNA template nor the ligation products were clearly observed by gel staining and would require indirect amplification methods for accurate detection. On the other hand, the double ligation product was not the only product of the reaction; the amount of double ligation product was approximately equal to single ligation products at both room temperature and 50 °C for rate limiting RNA template concentrations (1 and 0.2 X). This was unexpected and suggests templated ligation does not favour pseudo-unimolecular kinetics for the second ligation reaction. Previous studies have shown that artificial backbones generally destabilise duplex DNA.[28,29] As a consequence, we hypothesise that the first ligation either disrupts the hybridisation of the already hydridised strands to the template or that its formation interferes with the favourable orientation of the reactive groups for the second ligation reaction. In both cases, this would result in a second, more stringent rate-limiting ligation step. This perhaps explains the excellent specificity of dual ligation to its complementary strand (Fig. S9); even a single internal C-U mismatch in the hybridisation region is energetic costly and prevents formation of the double ligation product. Yet this may also explain the lower limit of detection of 0.3 attomoles in qPCR; the extremely low template concentrations mean the formation of a stable complex for the second ligation may be inhibited. Therefore, to reduce the limit of detection further may require new artificial backbone linkages or the introduction of duplex stabilizing modifications such as LNA.

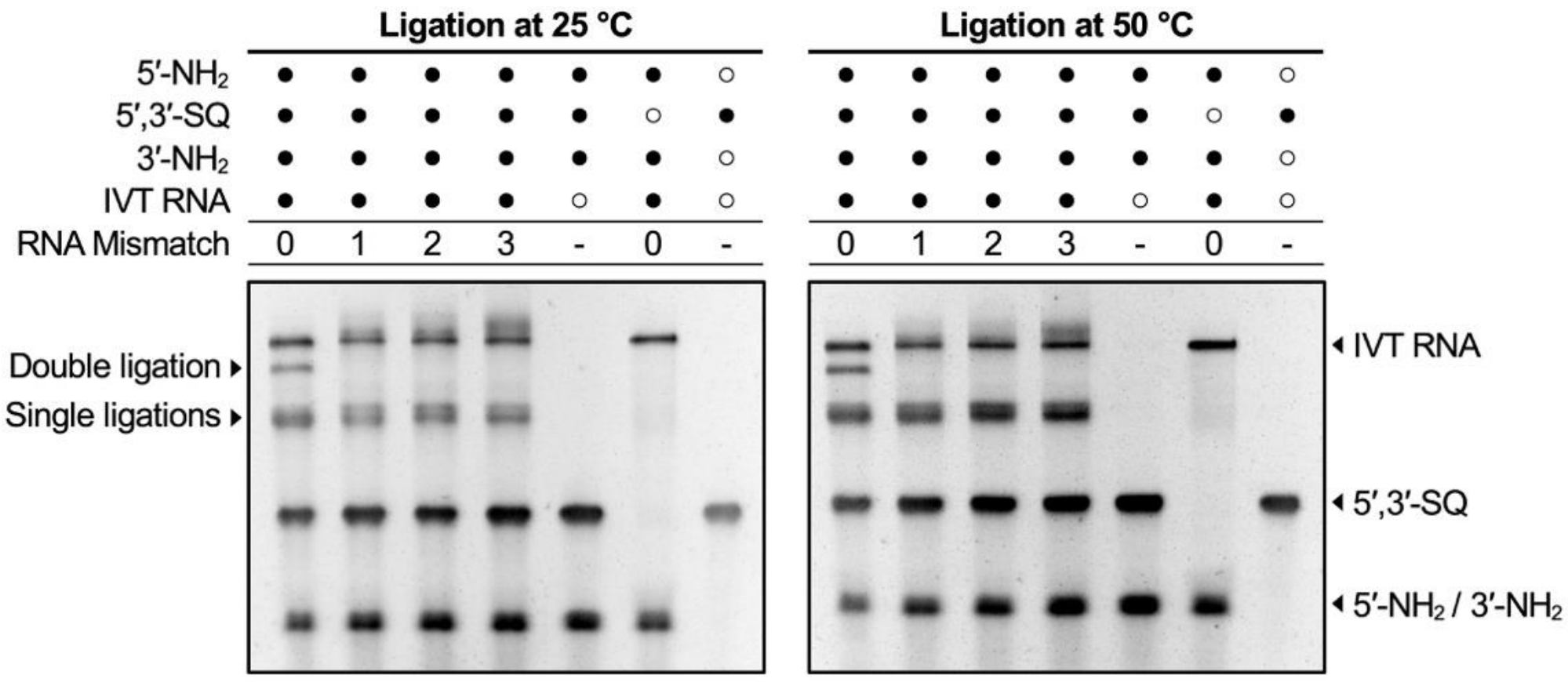

Fig. S9. Dual squaramide ligation products were formed only in the presence of perfectly complementary RNA targets at both room temperature (25 °C) and 50 °C. Reactions were performed with a 1:1:1:1 ratio 5',3'-di-squaramide (5',3'-SQ), 5'-amino (5'-NH_2), 3'-amino (3'-NH_2) oligonucleotides and IVT RNA target (9 picomoles each) at 25 or 50 °C for 15 min. The number of mismatches in the target RNA are indicated in the figure and the exact sequences can be found in in Supplementary Table S4. Reactions were analysed on 15 % denaturing polyacrylamide gels post-stained with RedSafe dye. Black circles indicate a component was added to the reaction lane, while hollow white circles indicate the component was omitted. The oligonucleotides and templates for RNA generation can be found in Supplementary Table S4.

3. In Fig. 2A, the NH_2 group on the first yellow chain is missing.

This has been fixed.

4. In Fig. 3C, the x-axis should represent time, not temperature.

This is a good spot. We have updated the axis with the correct label.

Reviewer 2.

1. The authors accused reverse transcriptase for being biased during introduction. Though this is true, part of the problem roots from the choice of reagent or strategy. This is intrinsic for every kind of assay, including the study being reviewed here […] Therefore, within the scope of this manuscript, chemical ligation demonstrated no advantage over reverse transcription regarding "bias" or any other metrics.

We apologise for misguiding the reviewer in the introduction. We focussed on highlighting the limitations of enzymatic methods but failed to do so for chemical ligation too. As a result, we failed to highlight the one of the key goals of the manuscript – to demonstrate chemical ligation is even a feasible alternative. As the reviewer points out at the end: *"In summary, the authors reported an RNA qPCR strategy using chemical ligation. The strategy works, but demonstrating no advantage over current [enzymatic] strategies."* This is what we too want to highlight – the strategy is feasible and reaches levels comparable to enzymatic methods.

Numerous chemical ligation systems have been reported for nucleic acids that generate artificial linkages over the last 30 years. Few are compatible with polymerase-based amplification and fewer still are easily prepared without extra purification before amplification. As a result, to the best of our knowledge, there are no examples in literature that even try to compete with enzymatic methods in very sensitive, quantitative detection applications such as qPCR detection of RNA. Thus, our work

represents a significant step forward in the field of chemical ligation reactions even if it does not immediately demonstrate better performance than enzymatic methods.

We have revised the introduction as follows:

> Pure chemical ligation has been used to link oligonucleotides to form canonical or modified backbones. Of the wide range of reactions possible,[12] only a handful of linkages (amide,[13] phosphate,[14] phosphorothioate,[15–17] phosphoramidate,[18–21] triazole[22,23] and urea[24]) can be read-through by polymerases. Of these linkages, only several can be prepared *in situ* without the need for further purification before amplification by polymerases.[15–17,20,21] Of these reactions, the reagents for the reaction have poor stability in solution[15,20] or the reaction rate is very slow (hours).[16,17,21] As a result, while chemical ligation reactions theoretically offer benefits over enzymatic RNA to DNA conversion, to date none have been practically used as even alternatives to enzymatic methods in sensitive, quantitative applications such as qPCR.
>
> To this end, a promising chemical ligation system is squaramide formation. […]
>
> Herein, we demonstrate un-templated ligation limits the sensitivity of chemical ligation and explore strategies to overcome this hurdle. In doing so, we show chemical ligation is a feasible alternative to enzymatic methods in quantitative applications such as qPCR detection of RNA. Through careful design and optimization sub-attomoles of long ~2,500-nt RNAs are quantitatively detected in complex systems by squaramide ligation. […]

as well as the conclusion:

> In summary, we have demonstrated squaramide ligation reactions can convert a target RNA sequence to DNA in a complex mixture of RNAs for quantitative PCR detection. However, un-templated ligation limits the sensitivity of method and results in false positives at very low target RNA concentrations. To overcome this limitation, we developed a dual ligation system, which requires two independent chemical ligation reactions to happen to generate DNA products that is amplifiable. In the absence of a template RNA, this reaction is extremely unfavourable and effectively removes the false positives when coupled with probes for specific amplicon detection. This strategy is in principle extendable to other chemical ligation systems.
>
> For squaramide ligation in particular, the reaction is simple to setup […]

2. The manuscript lacks direct comparison with enzymatic reaction (reversed transcription or ligase).

We thank the reviewer for this good point. We have now performed reverse transcription qPCR using our molecular beacons conditions for mRNA site 1. This provides a direct comparison to what we feel are the optimal conditions reported in the manuscript for squaramide ligation. Our data shows reverse transcription qPCR can detect RNA to at least one order of magnitude lower concentration. This has been incorporated as follows (methods omitted here for brevity):

> It is interesting to note that we were unable to detect concentrations lower than 0.3 attomoles (0.0003 femtomoles), while on the other hand RT-qPCR could reliably detect 0.03 attomoles using the same Molecular beacon detection chemistry (Fig. S8) and in theory go to single molecule yoctomole limits.

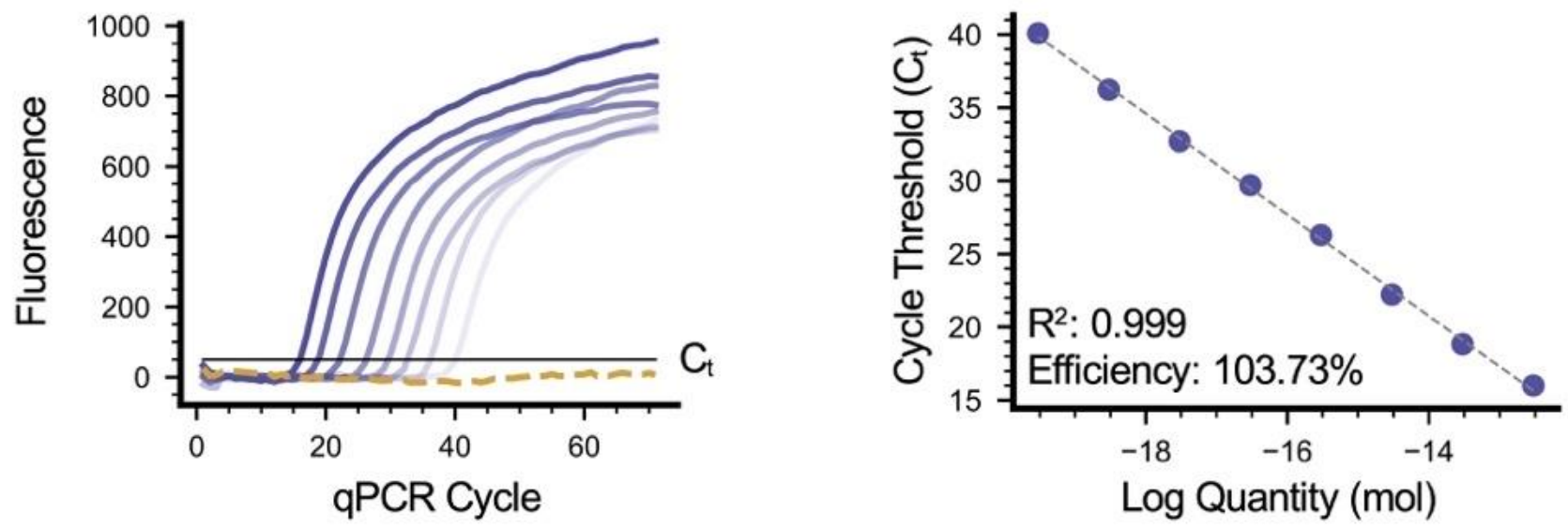

Fig. S8. mRNA target site 1 conversion of RNA to cDNA using reverse transcription followed by Molecular Beacon qPCR. Reverse transcription was performed with target RNA (300, 30, 3, 0.3, 0.03, 0.003, 0.0003, 0.00003 femtomoles) in the presence of 100 ng total RNA for 120 min at 37 °C. Dashed yellow lines are no template controls. Solid horizontal black lines are the threshold fluorescence for detection (50 RFU). Primers used are listed in Supplementary Table S2.

3. The claim of limit of detection by the authors is not convincing. The calculation using no-template sample C_t value is problematic, particularly for EvaGreen qPCR. In Figure 2B, no-template sample C_t values were way over 30, making the values not reliable. Very high C_t values suggest a few copies before qPCR, making the amplification success rate during the first few cycles Boolean. Figure 3B and 3C are good examples. Furthermore, the no-template samples gave actual C_t values.

We apologise about the lack of clarity here. Until Figure 4, we merely use the difference between the no template control and positive amplification as an approximation to guide optimisation of the reaction. We mention this when first introducing this equation:

> For optimization purposes, we assumed that qPCR amplification and ligation efficiency are both quantitative. Therefore, comparison of the qPCR C_t value with and without the RNA target allows approximation of the limit of detection using the equation $\mathrm{RNA_{in}} \times 2^{\mathrm{Ct\,(RNA)-Ct\,(NTC)}}$, where RNA_{in} is the initial quantity of RNA used in the template containing reaction

but failed to emphasise the approximate nature of the values in subsequent optimisation figures and related text. We have gone through and revised the manuscript to highlight that this is a guide in optimisation of the assay until Figure 4 results (added hypothetical before any such values):

> For the 3'-pre-activated mono-squaramide system (Figure 1, pink), the hypothetical limit of detection was ~0.5 femtomoles, which is consistent with our previous results.[24] For the *in-situ* system (Figure 1, green), the hypothetical limit of detection was ~0.2 femtomoles.
>
> **Figure 2.** Dual squaramide ligation performed using mono-squarate pre-activated oligonucleotides (external = brown, internal = yellow) perform better than one-pot (grey) reactions for dual squaramide ligation. A) provides an overall schematic of the various ligation variations. B) demonstrates the theoretically wide detection range provided by pre-activated system (hypothetically 0.3 attomoles based on the equation $RNA_{in} \times 2^{Ct\,(RNA)-Ct\,(NTC)}$ where RNA_{in} is 300 femtomoles).
>
> To evaluate this dual ligation system, we again compared C_t values for ligation reaction with and without a target RNA using qPCR. When ligation was performed in situ with squarate ester (Figure 2B), the hypothetical limit of detection was improved from ~0.2 to ~0.05 femtomoles. Further optimization of the squarate ester concentration in the *in-situ* reaction (5 to 0.04 mM) failed to improve the hypothetical detection limit as the on-target ligation efficiency significantly dropped (Fig. S1). However, the true strength of this design was revealed when the central di-amino oligonucleotide was pre-activated (Figure 2B); hypothetical detection limits of ~0.0007 femtomoles or ~0.7 attomoles appeared feasible. […] Furthermore, there was no benefit to activating the two

terminal amino oligonucleotides instead of just the central di-amino oligonucleotide, with hypothetical detection limits remaining at ~0.4 attomoles.

We also note that for Figure 3, we never claim any changes in limit of detection. This figure more serves to show no changes in efficiency are observed as a function of ligation temperature or time before qPCR detection.

4. In Figure 4A, the signal of 0.003 fmoles sample was very close to the no-template sample. Imagine a real sample giving out such a curve, it would be difficult to claim a positive detection.

We thank the reviewer for this fair and important comment. While it is detectable, it is very close to the NTC C_t value. For EvaGreen chemistry, we have revised this value to 0.03 fmol, which is greater 3.3 cycles away from the NTC C_t value.

5. Figure 4B using molecular beacon presented much reliable demonstration of limit of detection. Nevertheless, typical RT-qPCR achieves much lower limit of detection (a few dozens of molecules), out-performing the strategy reported here.

Figure 4B is the culmination of our optimisation and represents what we believe to be the true limit of detection of our optimised conditions (0.3 amol). We appreciate the reviewer is convinced by this value.

As stated earlier, our chemical ligation method reaches limits of detection comparable to enzymatic methods, which brings chemical ligation systems far closer to be a feasible alternative than was previously possible.

6. The problem of Figure 4B is the assay stability. The C_t values of 10-fold diluted samples were not uniformly distributed. The instability may root from low ligation efficiency.

We have repeated the assay multiple times and the ΔC_t for 10-fold dilutions is 3.66 ± 0.15, for molecular beacon mRNA target site 1 (n = 3, 110.0 ± 4.6 % amplification efficiency), and 3.37 ± 0.11 for molecular beacon mRNA target site 2 (n = 3, 101.0 ± 3.3 % amplification efficiency). These values are slightly on the higher side of 3.33 for perfect amplification efficiency but are still reasonable. Note that all of our repeats are completely independent runs.

7. Given the number of input molecules, the qPCR C_t values should be much lower than what Figure 4A shows, even considering the 100-fold dilution. For example, 0.003 fmoles after 100-fold dilution should be roughly 1E4 molecules, not concordant with C_t value of 30.

We have now performed RT-qPCR (Fig. S8) using identical chemistry (Molecular Beacons) and instrument setup (excitation and emission filters) to squaramide ligation qPCR (Fig. 4B / Fig. S6 for all repeats) and the C_t values are in a comparable range suggesting squaramide ligation does not cause unexpected changes. We note the Molecular Beacon C_t values are lower than EvaGreen purely due to the probes allowing only 1 (partially quenched) fluorophore per target cf. multiple intercalating dyes in the case of EvaGreen. In our hand and equipment setup, we believe the C_t values seem reasonable for EvaGreen (Fig. 4A).
