# Supplementary material for: Non-enzymatic conversion of RNA sequence information into DNA by squaramide ligation for accurate RNA quantification
Source: Commun Chem. 2026 May 19;9:272. doi: 10.1038/s42004-026-02069-5 (PMC13454357; doi:10.1038/s42004-026-02069-5)
Supplement: Supplementary file 2 [file 42004_2026_2069_MOESM2_ESM.pdf]

Non-enzymatic conversion of RNA sequence information into DNA by squaramide ligation for accurate RNA quantification

Lapatrada Taemaitree,[1,2] Friedrich Burba,[1] Nina Harvey,[3,4] Arun Shivalingam,[1] Tom Brown,[1] Afaf H. El-Sagheer[1,3,4]*

[1] Department of Chemistry, University of Oxford, Chemistry Research Laboratory, 12 Mansfield Road, Oxford, OX1 3TA, UK

[2] Department of Integrated Science, Faculty of Science, Khon Kaen University, Khon Kaen 40002, Thailand

[3] School of Chemistry and Chemical Engineering, University of Southampton; Highfield, Southampton, SO17 1BJ, United Kingdom

[4] Institute for Life Sciences, University of Southampton; Highfield Campus, Southampton, SO17 1BJ, United Kingdom

* To whom correspondence should be addressed: Email: ahes@soton.ac.uk

Supplementary Information

Methods

Oligonucleotide synthesis. DNA synthesis was performed on an Applied Biosystems 394 automated DNA/RNA synthesizer using a standard phosphoramidite cycle of detritylation, coupling, capping and oxidation using TCA (3% in dichloromethane), 1H-tetrazole (0.45 M in acetonitrile), Cap A (10% acetic anhydride, 10% lutidine and 80% tetrahydrofuran) / Cap B (16% N-methylimidazole in tetrahydrofuran) and iodine (0.02 M in tetrahydrofuran, pyridine and water) on a 0.2 or 1.0 µmole scale. Pre-packed nucleoside SynBase™ CPG 1000/110 (Link Technologies) resins, or 3'-amino-dT CPG (Cambio, cat. no. 20-2981-01) / 3'-dabcyl CPG (Cambio, cat. no. 20-5912-01) packed into a twist column (Glen research) were used for synthesis β-cyanoethyl protected phosphoramidites (dA-bz, dG-ib, dC-bz and dT where bz = benzoyl and ib = *iso*-butyryl, Sigma-Aldrich) were dissolved in anhydrous acetonitrile (0.1 M) immediately prior to use. The coupling time for dA, dC, dG and dT monomers was 45 s, and 10 min for modified monomers (5'-amino-dT, Cambio, cat. no. 10-1932-90; 5'-Fluorescein, Link Technologies, cat. no. 2134).

For terminal 3'-amino oligonucleotides, pre-packed 3'-amino-dT CPG resin (Cambio, cat. no. 20-2981-01) was used and coupled to standard phosphoramidite monomers. For terminal 5'-amino oligonucleotides, the last step of oligonucleotide synthesis involved coupling of a 5'-amino-dT phosphoramidite (Cambio, cat. no. 10-1932-90) for 10 min with no subsequent capping. The structure of the resin and phosphoramidite are shown below:

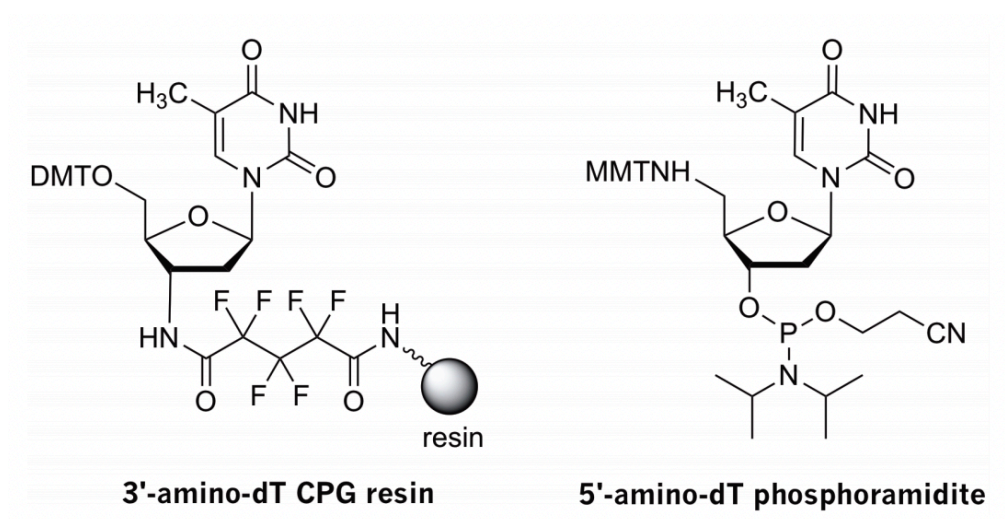

Stepwise coupling efficiencies were determined by automated trityl cation conductivity monitoring and were >98% in all cases.

Oligonucleotide deprotection. Unmodified oligonucleotides were cleaved from solid support and deprotected by exposure to a concentrated solution of aqueous ammonia in a sealed vial for 5 h at 55 °C. After drying in vacuo, oligonucleotides were dissolved in water and subject to further purification. Amino-modified oligonucleotide deprotection and cleavage from the resin was slightly altered to improve yields and purity as suggested by the supplier.

For terminal 3'-amino oligonucleotides, the resin was incubated with a concentrated solution of aqueous ammonia in a sealed vial for 18 h at 55 °C. After drying in vacuo, oligonucleotides were dissolved in water and RP-HPLC purified as described in the General Oligonucleotide Purification section.

For terminal 5'-amino oligonucleotides, prior to cleavage from the resin, the solid support was treated with diethylamine (20% in anhydrous acetonitrile) for 10 min at room temperature to suppress the formation of cyanoethyl adducts. The resin was then washed with acetonitrile (3 x 1 mL) and dried with argon before exposure to a solution (100 μL) of water in methanol (1:4 v/v) containing sodium hydroxide (0.4 M final concentration) for 3 h at 55 °C. This was to reduce acetylation of the amino groups during deprotection and cleavage of the oligonucleotide from the solid support. After cooling to room temperature, the solution was diluted with 9 volumes of water and desalted and RP-HPLC purified as described in the *Oligonucleotide Purification* section.

Oligonucleotide purification. *Desalting*. NAP™-10 columns (G.E. Healthcare Life Sciences, cat. no. GE17-0854-02) were used according to the manufacturer's instructions.

*RP-HPLC***.** Oligonucleotides were purified using a Gilson HPLC system with ACE® C8 column (10 mm x 250 mm, pore size 100 Å, particle size 10 μm) with a gradient of buffer A (0.1 M TEAB, pH 7.5, where TEAB = triethylammonium bicarbonate) to buffer B (0.1 M TEAB, pH 7.5 containing 50% v/v acetonitrile) and flow rate of 4 mL/min. For amino- and squaramide-modified oligonucleotides, a gradient of 15–35% buffer B was used with a total run time of 20 min. The TEAB buffer and acetonitrile in the collected fractions were removed *in vacuo* or by freeze drying.

Oligonucleotide mass spectrometry. All oligonucleotides were characterised by negative-mode electrospray using a UPLC-MS Waters XEVO G2-QTOF mass spectrometer and an Acquity UPLC system with a BEH C18 1.7 μm column (Waters). A gradient of methanol in triethylamine (TEA) and hexafluoroisopropanol (HFIP) was used (buffer A, 8.6 mM TEA, 200 mM HFIP in 5% methanol/water (v/v); buffer B, 20% v/v buffer A in methanol). Buffer B was increased from 0–70% over 7.5 min or 15–30% over 12.5 min for normal oligonucleotides and 50–100% over 7.5 min for hydrophobic oligonucleotides. The flow rate was set to 0.2 mL/min. Raw data were processed and deconvoluted using the deconvolution software MassLynx v4.1.

Target RNA *in vitro* transcription for qPCR. A T7 promoter-containing plasmid pcDNA3.1-hAsCpf1 (Addgene, plasmid number #69982, kindly gifted by Dr. Feng Zhang) was used as the DNA template for *in vitro* transcription (IVT) of a 2,665-mer RNA. Prior to IVT, the plasmid (1 μg/μL,

5 µL) was linearised using PmlI restriction enzyme (2 U/µL, 2.5 µL, NEB, cat. no. R0532S) in CutSmart buffer (10x, 5 µL, NEB) and water (37.5 µL) for 1 h at 37 °C. The enzyme was then inactivated by heating the sample (20 min, 65 °C). The RNA was prepared using the MEGAScript™ T7 *in vitro* transcription kit (Thermo Fisher Scientific, cat. no. AM1334). One reaction contained ATP (75 mM, 2 µL), CTP (75 mM, 2 µL), GTP (75 mM, 2 µL), UTP (75 mM, 2 µL), T7 enzyme mix (2 µL), reaction buffer (10x, 2 µL), linearised plasmid or DNA template (5 or 1 µL) and water (up to total volume 20 µL). After incubation for 16 h at 37 °C, the transcription reaction was treated with Turbo DNase (2 U/µL, 1 µL) for 1 h at 37 °C. Next, the transcribed RNA was purified using the MEGAClear™ transcription Clean-up kit (Thermo Fisher Scientific, cat. no. AM1908). The transcription reaction was mixed with Elution Solution (80 µL), Binding Solution (350 µL) and ethanol (250 µL) and subsequently transferred to the filter cartridge. Following centrifugation (14,000 *x g*, 1 min), the flow-through was discarded and the sample was washed using Wash Solution (2 x 500 µL). Next, Elution Solution (100 µL) was added to the filter cartridge and heated for 10 min at 70 °C. After centrifugation (14,000 *x g*, 1 min), the eluted RNA was collected and the concentration was measured using a NanoDrop™ 2000/2000c Spectrophotometer (Thermo Fisher Scientific) prior to characterisation on a 1% agarose gel stained with 0.5x SYBR gold (1x TBE running buffer, 126 V).

Reverse transcription. As a control for squaramide ligation, IVT mRNA was converted cDNA using a High-capacity cDNA kit (Thermo Fisher Scientific, cat. no. 4368814). One reaction contained RT Buffer (10x, 2 µL), dNTPs (25x, 100 mM, 0.8 µL), random primers (10x, 2 µL), MultiScribe Reverse Transcriptase (50 U/µL, 1 µL), total RNA (100 ng/µL, 1 µL), Murine RNase inhibitor (4 U/µL, 1 µL, NEB, cat. no. M0314S), IVT mRNA template (appropriate 50x target concentration, 1.35 µL) and water (up to 20 µL). Reactions were then incubated at 25 °C for 10 min, at 37 °C for 120 min and finally at 85 °C for 5 min to inactivate the enzyme. The reserve transcription mixture was diluted 50-fold prior to qPCR.

Total RNA preparation. Total RNA was extracted from MCF-7 cells (cultured in DMEM supplemented with 10% v/v FBS) using the PureLink® RNA Mini kit (Thermo Fisher Scientific, cat. no. 12183018A) according to manufacturer's instructions. After cell dissociation using enzyme-free Hank's Balanced salt solution (Thermo Fisher Scientific, cat. no. 13150016), 1–5 x 10^6 cells were transferred to 50 mL falcon tube and centrifuged (2,000 *x g*, 5 min, 4 °C). The media was discarded and the cell pellet was washed with PBS buffer. After centrifugation (2,000 *x g*, 5 min, 4 °C), the PBS buffer was removed and the pellet resuspended in Lysis Buffer (600 µL, containing 1% v/v 2-mercaptoethanol) by vigorous vortexing. The dispersed and lysed cell pellet was then homogenised by passing the cell lysate through a 21-gauge needle attached to a syringe (10 times). Next, an equal volume of 70% ethanol was added to the cell homogenate. After vortexing thoroughly, the

mixture (700 µL) was transferred to the Spin Cartridge, centrifuged (12,000 *x g*, 15 s) and the flow-through was discarded. The process was repeated until no cell mixture remained. Wash Buffer I (350 µL) was then added to the Spin Cartridge. After centrifugation, the flow-through was discarded and on-column DNase digestion was performed by applying the DNase mixture containing Turbo DNase (2 U/µL, 15 µL), DNase I reaction buffer (10x, 8 µL, NEB) and RNase-free water (57 µL) onto the Spin Cartridge membrane before incubation for 15 min at room temperature. Three more rounds of washing were performed using Wash Buffer I (1 x 350 µL) and Wash Buffer II (2 x 500 µL). After further centrifugation (12,000 *x g*, 1 min) to remove residual liquid, RNA was eluted by adding RNase-free water (100 µL) to the Spin Cartridge, incubating for 1 min at room temperature and centrifugation (12,000 *x g*, 2 min). The concentration of total RNA was measured using a NanoDrop™ 2000/2000c Spectrophotometer (Thermo Fisher Scientific).

qPCR using EvaGreen. A polymerase master mix consisting of Vent (exo–) DNA polymerase (2 U/µL, 0.25 µL, NEB, cat. no. M0257S), ThermoPol buffer (10x, 2 µL), EvaGreen (20x, 1 µL, Biotium, cat. no. 31000), heat-activated CleanAmp® dNTPs (10 mM, 0.4 µL, tebu-bio, cat. no. 040N-9506-2) and water (13.35 µL) was prepared in the 0.2 mL white 8-tube PCR strips (Bio-rad, cat. no. TLS0851). The mix was then added to a solution of forward primer (10 µM, 1 µL), reverse primer (10 µM, 1 µL) and template (100-fold diluted samples from squaramide ligation, 1 µL). Total reaction volume is 20 µL. After sealing the PCR tubes with the compatible ultraclear 8-cap strips (Bio-rad, cat. no. TCS0803), the reaction mixture was mixed thoroughly by vortexing and centrifuged before qPCR amplification.

After initial thermal activation (120 s, 95 °C), 46 cycles of denaturation (15 s, 95 °C) and annealing/extension (60 s, 60 °C) were performed on the Bio-rad CFX96 qPCR instrument. At the end of each extension step, samples were excited at 450–490 nm and the emission at 510–530 nm was monitored. For melt curves analysis, samples were heated from 60 to 90 °C post-PCR with emission recorded every 0.5 °C using a ramp rate of 6 °C/min. Amplification curves were baseline corrected using the CFX96 internal analysis software and standard curves plotted using custom Python scripts.

qPCR using Molecular Beacons. A polymerase master mix consisting of Vent (exo–) DNA polymerase (2 U/µL, 0.25 µL, NEB, cat. no. M0257S), ThermoPol buffer (10x, 2 µL), heat-activated CleanAmp® dNTPs (10 mM, 0.4 µL, tebu-bio, cat. no. 040N-9506-2) and water (14.15 µL) was prepared in the 0.2 mL white 8-tube PCR strips (Bio-rad, cat. no. TLS0851). The mix was then added to a solution of molecular beacon, forward primer, reverse primer as described in the table

below and target RNA (100-fold diluted samples from squaramide ligation, 1 µL). Total reaction volume is 20 µL.

Molecular beacon	Forward primer	Reverse primer
10 µM, 0.3 µL (AA)	10 µM, 1.6 µL	10 µM, 0.3 µL

After sealing the PCR tubes with the compatible ultraclear 8-cap strips (Bio-rad, cat. no. TCS0803), the reaction mixture was mixed thoroughly by vortexing and centrifuged before qPCR amplification.

After initial thermal activation (120 s, 95 °C), 71 cycles of denaturation (15 s, 95 °C) and annealing/extension (60 s, 57.5 °C) were performed. At the end of each extension step, samples were excited at 450–490 nm and the emission at 510–530 nm was monitored. For melt curves analysis, samples were heated from 25 to 90 °C post-PCR with emission recorded every 0.5 °C using a ramp rate of 6 °C/min. Amplification curves were baseline corrected using the CFX96 internal analysis software and standard curves plotted using custom Python scripts.

qPCR using a TaqMan probe. A polymerase master mix consisting of Vent (exo–) DNA polymerase (2 U/µL, 0.25 µL, NEB, cat. no. M0257S), Taq DNA polymerase (5 U/µL, 0.1 µL, cat. no. M0273S) ThermoPol buffer (10x, 2 µL), dNTPs (10 mM, 0.4 µL) and water (14.15 µL) was prepared in the 0.2 mL white 8-tube PCR strips (Bio-rad, cat. no. TLS0851). The mix was then added to a solution of TaqMan probe, forward primer, reverse primer as described in the table below and target RNA (100-fold diluted samples from squaramide ligation, 1 µL). Total reaction volume is 20 µL.

TaqMan probe	Forward primer	Reverse primer
10 µM, 0.15 µL	10 µM, 0.6 µL	10 µM, 0.6 µL

After sealing the PCR tubes with the compatible ultraclear 8-cap strips (Bio-rad, cat. no. TCS0803), the reaction mixture was mixed thoroughly by vortexing and centrifuged before qPCR amplification.

After initial thermal activation (120 s, 95 °C), 45 cycles of denaturation (15 s, 95 °C) and annealing/extension (60 s, 57.5 °C) were performed. At the end of each extension step, samples were excited at 450–490 nm and the emission at 510–530 nm was monitored. Amplification curves were baseline corrected using the CFX96 internal analysis software and standard curves plotted using custom Python scripts.

Denaturing PAGE analysis of ligation products. Synthetic DNA templates were *in vitro* transcribed to generate RNA targets. The single stranded templates (1.25 µL, 100 µM, sequences listed in Supplementary Table S4) were mixed with the single stranded complementary T7 promoter (1.5 µL, 100 µM, sequence listed in Supplementary Table S4), annealing buffer (1 µL, 10x consisting of 100 mM Tris-HCl pH 8.5, 10 mM EDTA, 500 mM NaCl) and water (to a total volume of 10 µL). 0.8 µL of this mixture was used for in vitro transcription as described in **Target RNA *in vitro* transcription** section. Ligation reactions were then performed as described in the main manuscript methods section using the sequences in Supplementary Table S4. Reactions were loaded on 15 % denaturing polyacrylamide gels and run at 5 W until the loading dye reached the end of the gel. Gels were then post-stained with 1× Vivantis RedSafe nucleic acid stain and visualised using a SynGene Gel Documentation system.

Since an equal amount of ligation oligonucleotides were used (9 pmol), conversion to double and single ligation products was determined by dividing the intensity of the product band by the intensity of all other DNA oligonucleotide bands. This data analysis was performed using ImageJ, where the baseline was determined by the first centimeter near the well per lane and band positioning between lanes kept identical for integration of the area under the curve for each band.

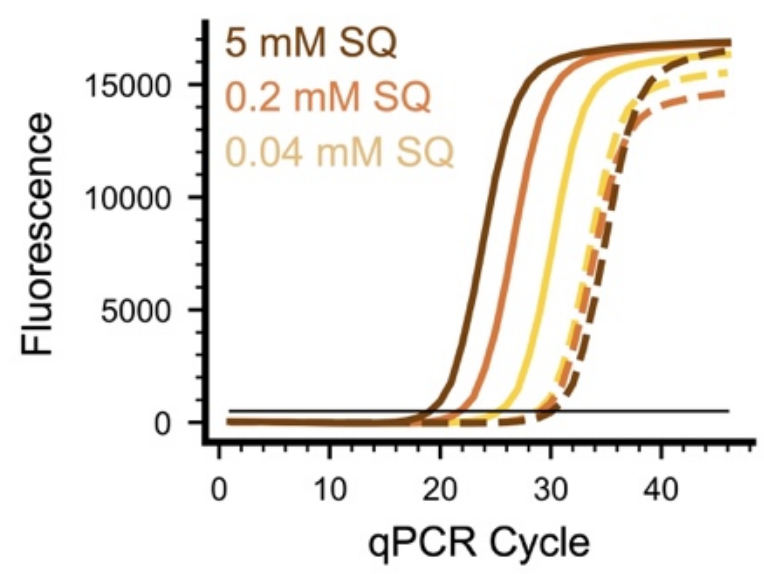

Supplementary Figure S1. Optimisation of squarate ester concentration for *in situ* dual ligation reaction systems. As the concentration of squarate ester decreases on-target ligation in the presence of 300 femtomoles RNA target decreases while off-target amplification in the absence of the RNA template remains relatively constant. Ligations were performed at room temperature for 15 min and analysed by EvaGreen qPCR. The oligonucleotides used are listed in Supplementary Table S2.

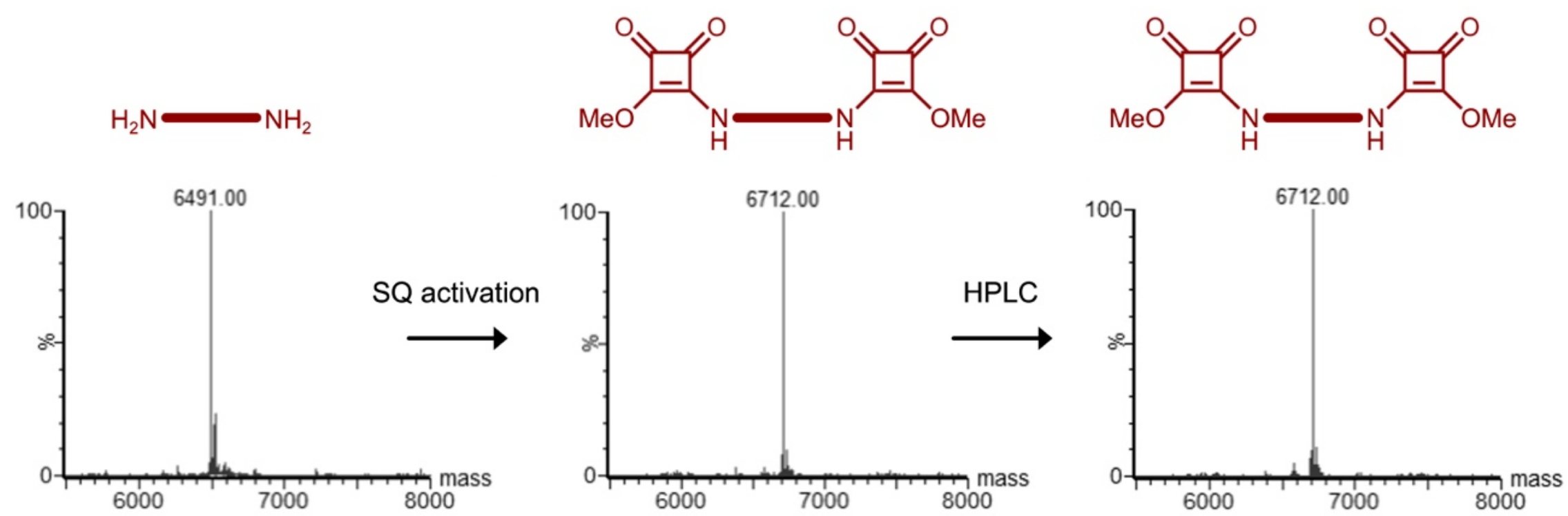

Supplementary Figure S2. UPLC-MS of di-squaramide pre-activated oligonucleotide (*DL1-Am5-Am3* and *DL1-Am5-Am3-SQ* in Supplementary Table S2). There is no cyclisation of the 5'-3'-terminal di-amino oligonucleotide and conversion to the pre-activated pre-cursors is quantitative. UPLC-MS of HPLC-purified mono-squaramide oligonucleotides demonstrate negligible degradation after purification in TEAB buffer, freeze-drying and resuspension in de-ionised water. Found masses are +/- 2 of expected masses.

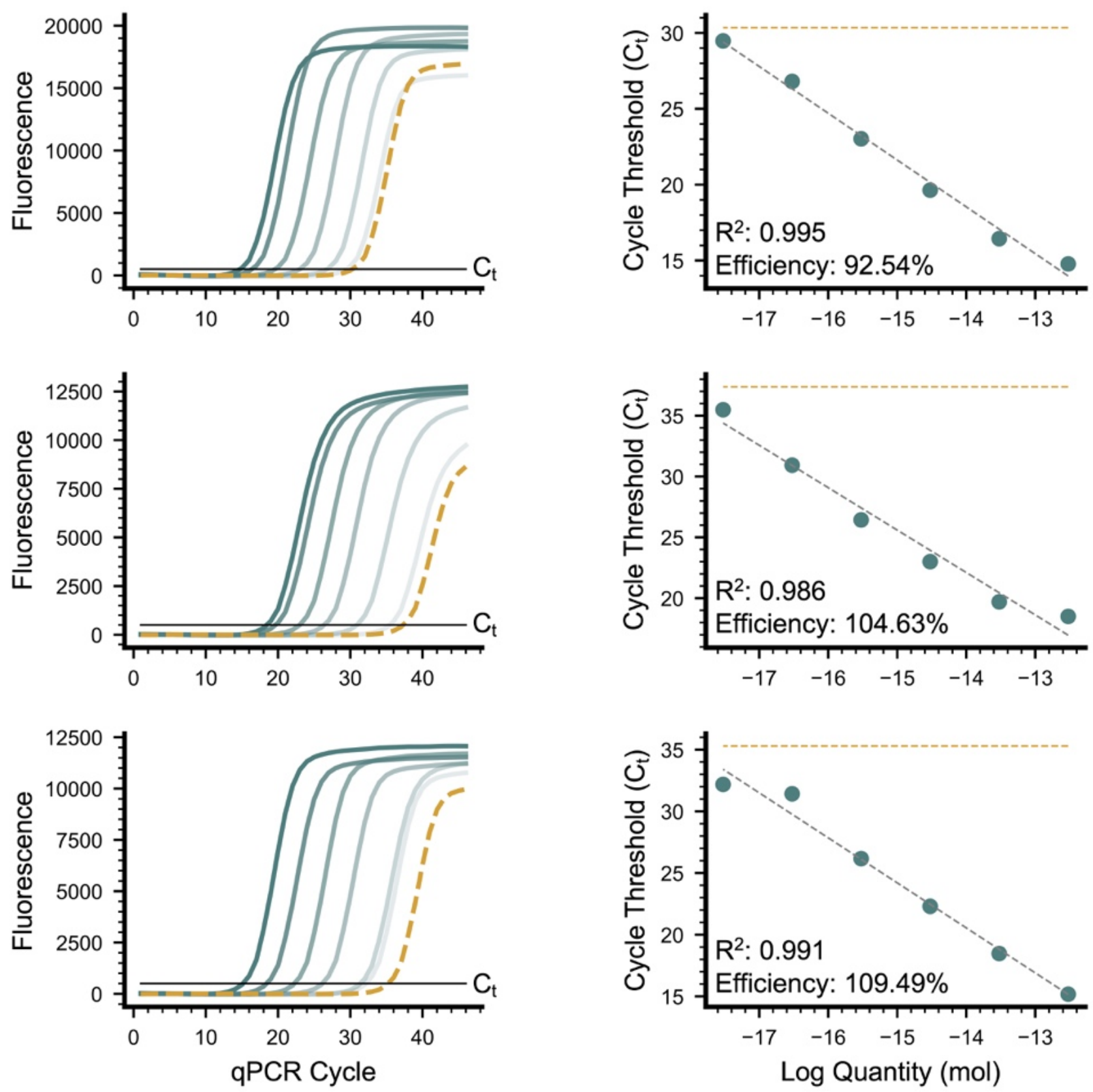

Supplementary Figure S3. mRNA target site 1 conversion of target RNA to DNA by squaramide ligation followed by EvaGreen qPCR. Reactions were performed with target RNA (300, 30, 3, 0.3, 0.03, 0.003 femtomoles) in the presence of 100 ng total RNA for 15 min at 50 °C. Each repeat is an independent run. Dashed yellow lines are no template controls. Solid horizontal black lines are the threshold fluorescence for detection (500 RFU). The oligonucleotides used are listed in Supplementary Table S2.

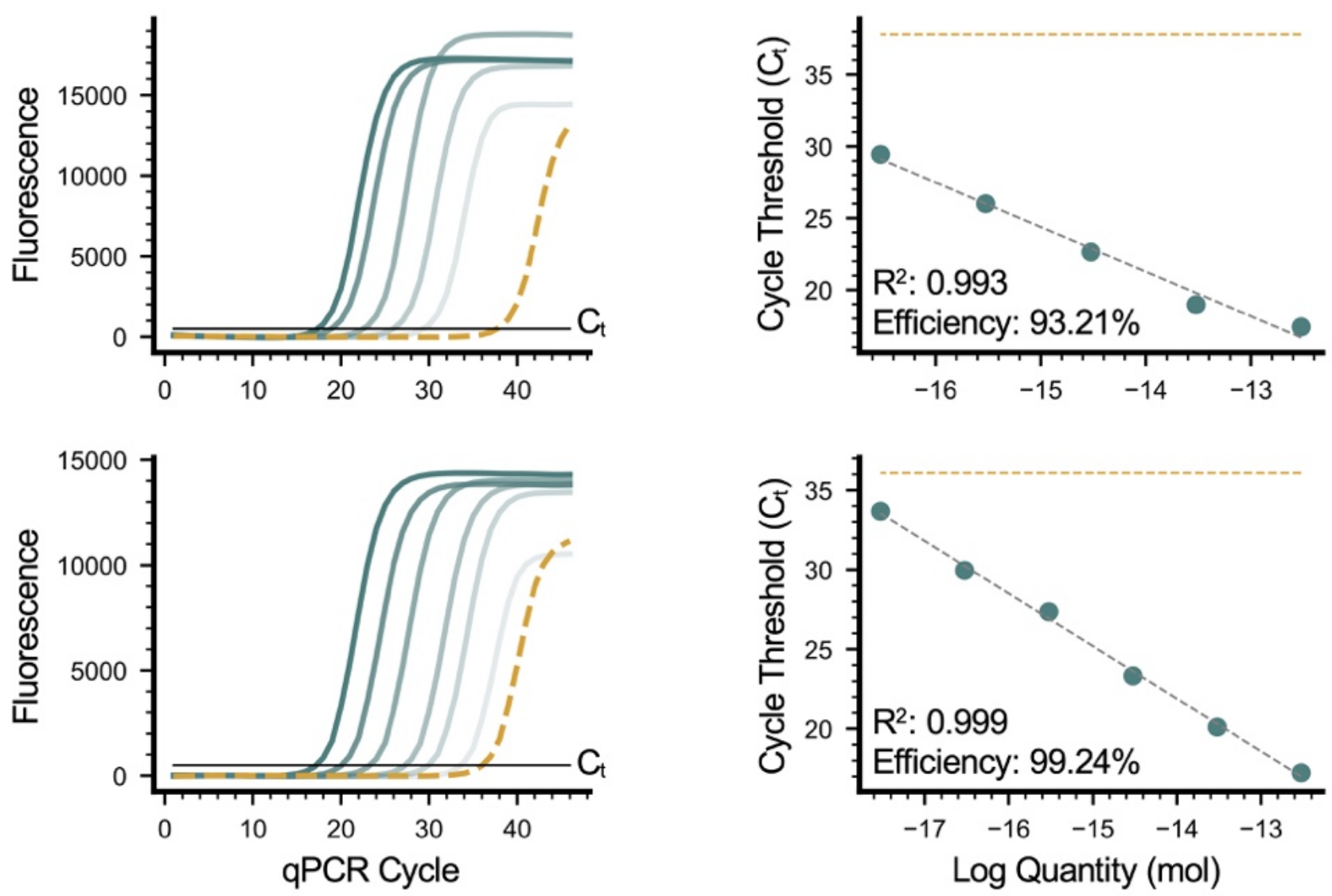

Supplementary Figure S4. mRNA target site 2 conversion of target RNA to DNA by squaramide ligation followed by EvaGreen qPCR. Reactions were performed with target RNA (300, 30, 3, 0.3, 0.03, 0.003 femtomoles) in the presence of 100 ng total RNA for 15 min at 50 °C. Each repeat is an independent run. Dashed yellow lines are no template controls. Solid horizontal black lines are the threshold fluorescence for detection (500 RFU). The oligonucleotides used are listed in Supplementary Table S3.

A

Cond.	[F]	[MB]	[R]
1	0.15	0.15	0.5
2	0.15	0.5	0.5
3	0.15	0.15	0.8
4	0.15	0.5	0.8
5	0.15	0.8	0.8
6	0.3	0.3	1.0
7	0.3	0.3	1.6

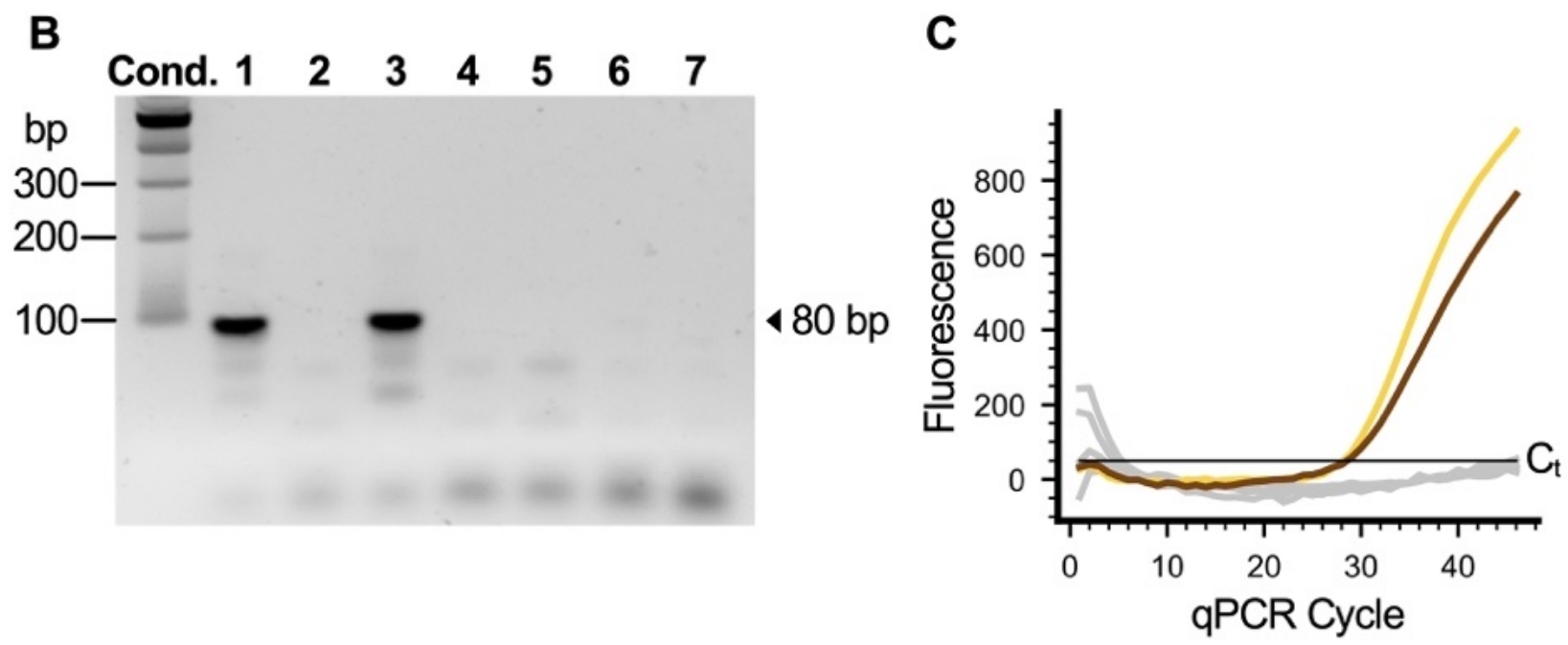

Supplementary Figure S5. Molecular beacon qPCR optimisation. A) shows the range of primer and probes concentrations evaluated. Of these conditions only 1 and 3 (those with the lowest molecular beacon concentrations) gave good amplification as evaluated by B) end-point agarose gel electrophoresis and C) qPCR. The PCR product was expected to be ~80 bp.

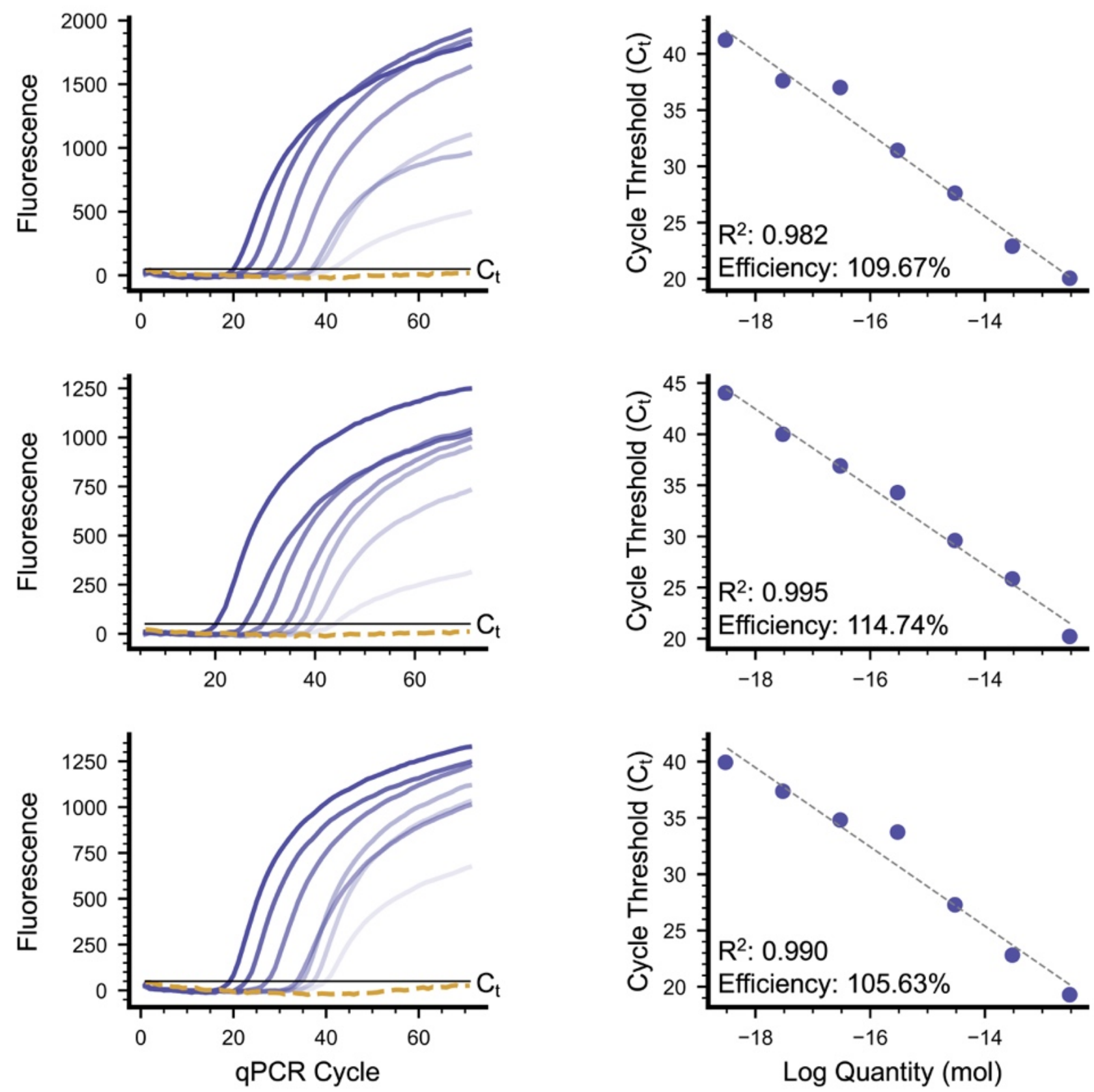

Supplementary Figure S6. mRNA target site 1 conversion of target RNA to DNA by squaramide ligation followed by qPCR using a Molecular Beacon. Reactions were performed with target RNA (300, 30, 3, 0.3, 0.03, 0.003, 0.0003 femtomoles) in the presence of 100 ng total RNA for 15 min at 50 °C. Each repeat is an independent run. Dashed yellow lines are no template controls. Solid horizontal black lines are the threshold fluorescence for detection (50 RFU). The oligonucleotides used are listed in Supplementary Table S2.

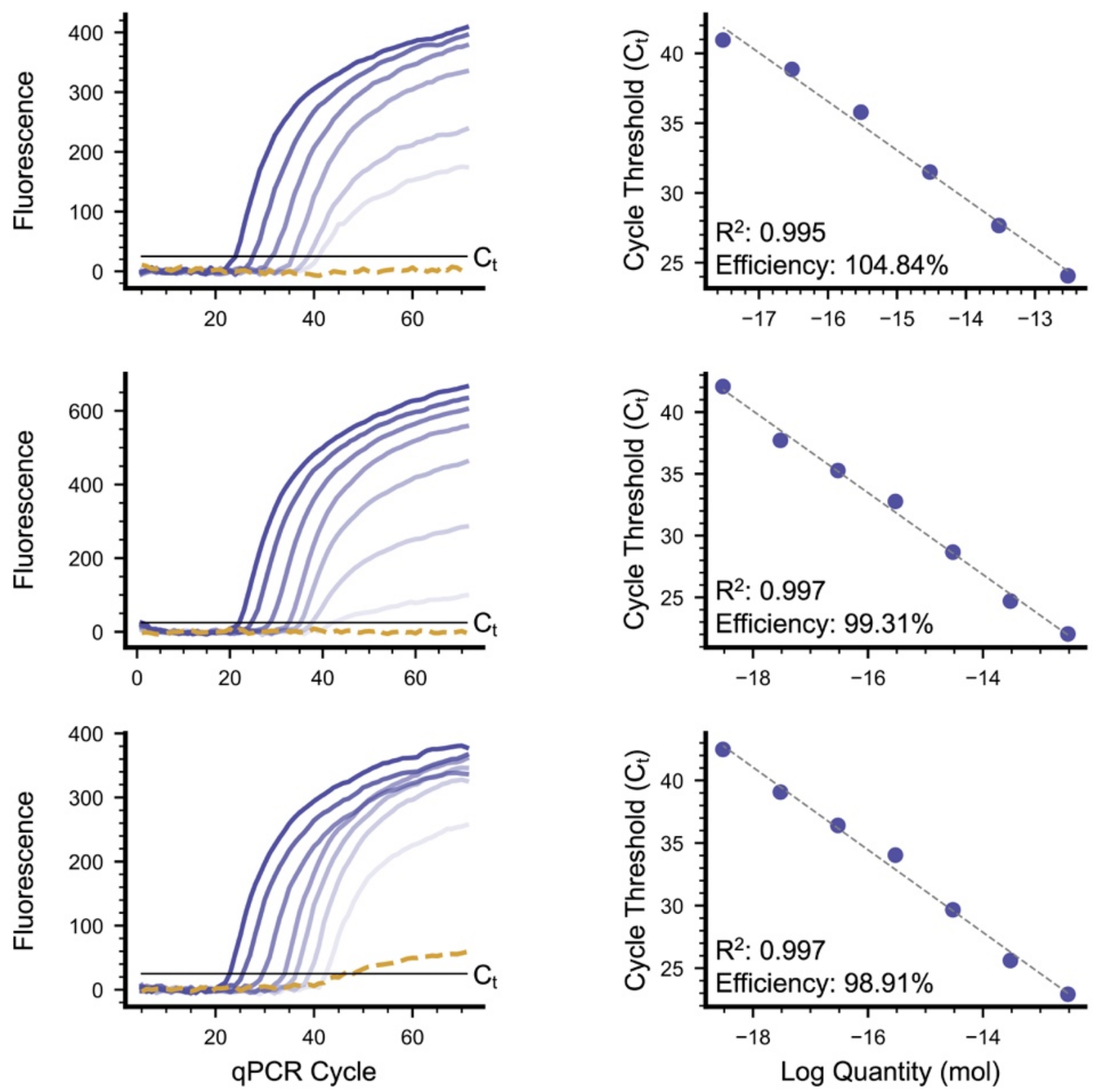

Supplementary Figure S7. mRNA target site 2 conversion of target RNA to DNA by squaramide ligation followed by Molecular Beacon qPCR. Reactions were performed with target RNA (300, 30, 3, 0.3, 0.03, 0.003, 0.0003 femtomoles) in the presence of 100 ng total RNA for 15 min at 50 °C. Each repeat is an independent run. Dashed yellow lines are no template controls. Solid horizontal black lines are the threshold fluorescence for detection (50 RFU). The oligonucleotides used are listed in Supplementary Table S3.

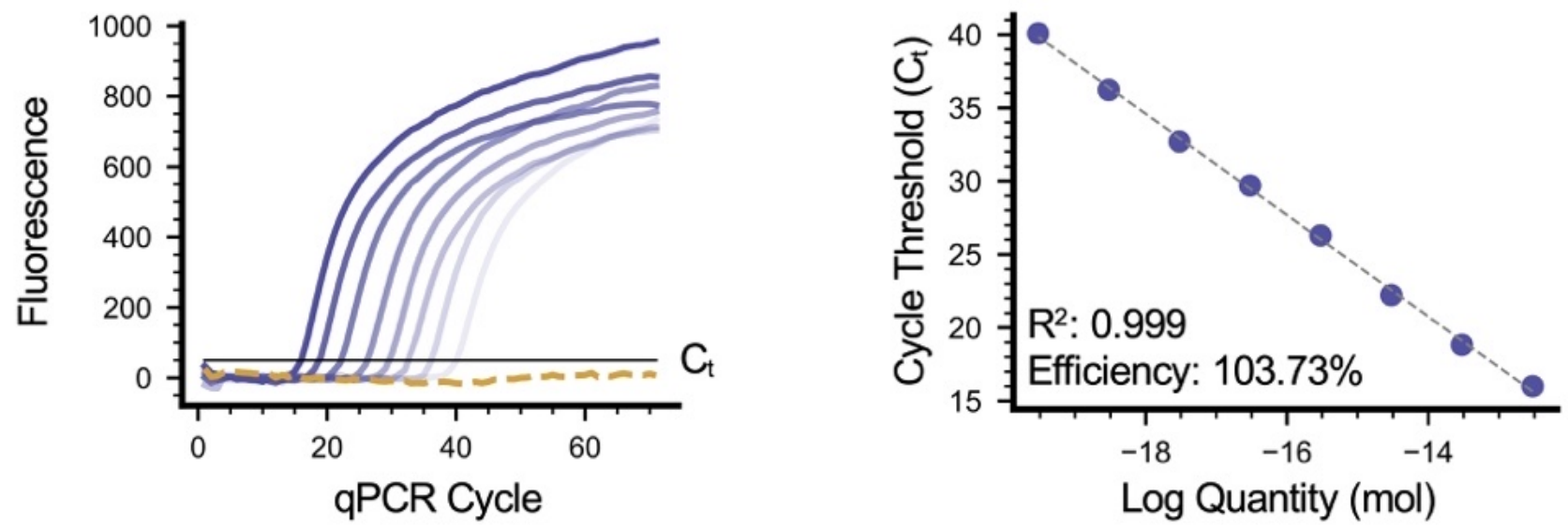

Supplementary Figure S8. mRNA target site 1 conversion of RNA to cDNA using reverse transcription followed by Molecular Beacon qPCR. Reverse transcription was performed with target RNA (300, 30, 3, 0.3, 0.03, 0.003, 0.0003, 0.00003 femtomoles) in the presence of 100 ng total RNA for 120 min at 37 °C. Dashed yellow lines are no template controls. Solid horizontal black lines are the threshold fluorescence for detection (50 RFU). Primers used are listed in Supplementary Table S2.

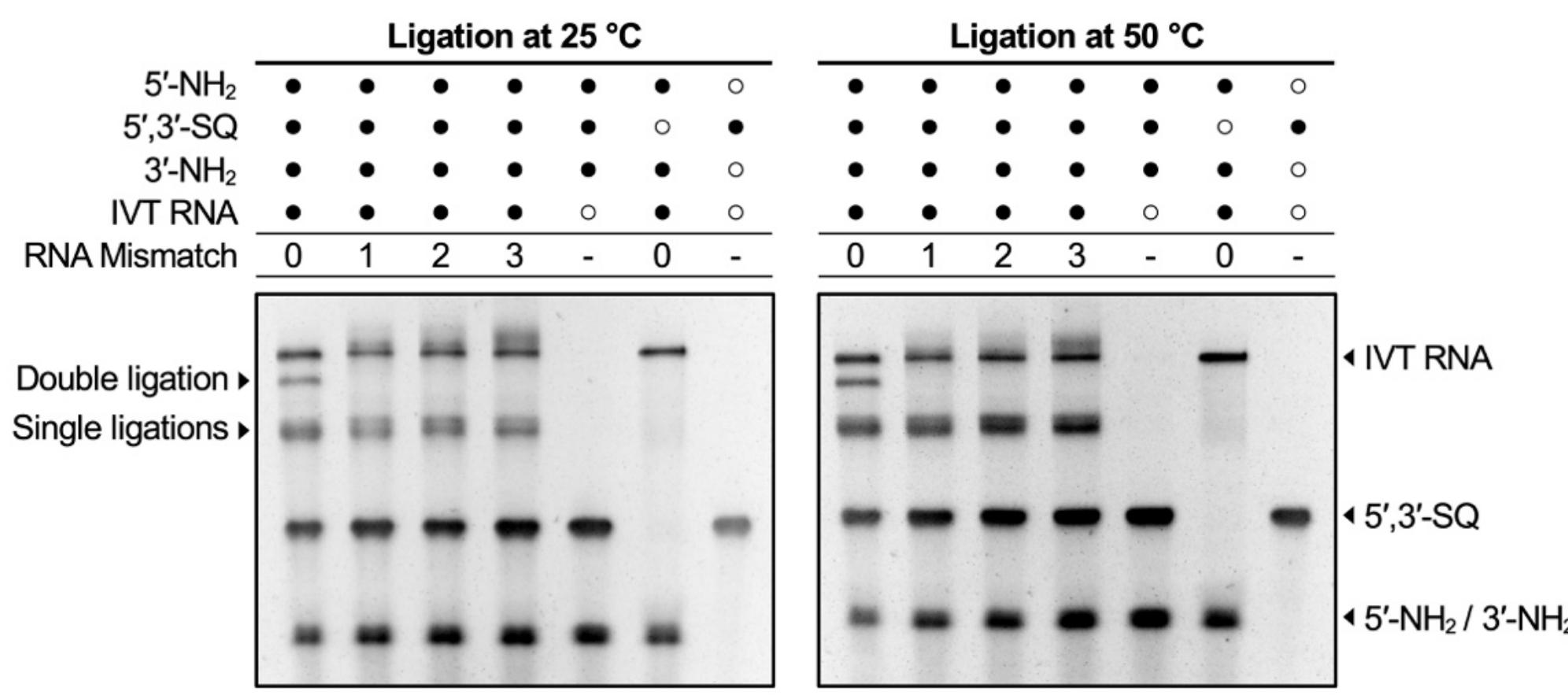

Supplementary Figure S9. Dual squaramide ligation products were formed only in the presence of perfectly complementary RNA targets at both room temperature (25 °C) and 50 °C. Reactions were performed with a 1:1:1:1 ratio 5',3'-di-squaramide (5',3'-SQ), 5'-amino (5'-NH_2), 3'-amino (3'-NH_2) oligonucleotides and IVT RNA target (9 picomoles each) at 25 or 50 °C for 15 min. The number of mismatches in the target RNA are indicated in the figure and the exact sequences can be found in in Supplementary Table S4. Reactions were analysed on 15 % denaturing polyacrylamide gels post-stained with RedSafe dye. Black circles indicate a component was added to the reaction lane, while hollow white circles indicate the component was omitted. The oligonucleotides and templates for RNA generation can be found in Supplementary Table S4. The uncropped gels are in Supplementary Figure S10 (C and D respectively).

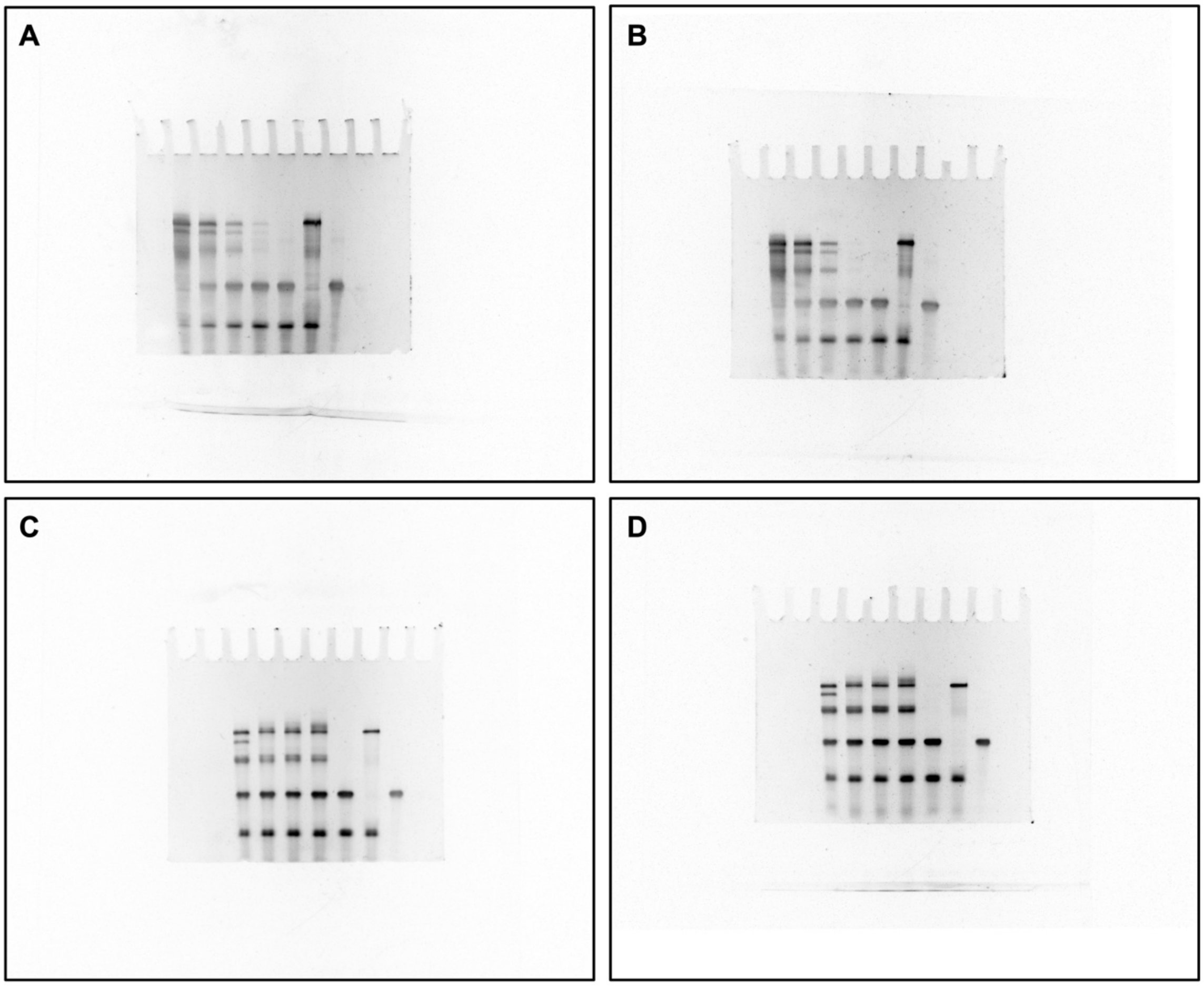

Supplementary Figure S10. Raw uncropped gels for Figure 5 (A and B) and Supplementart Figure S9 (C and D).

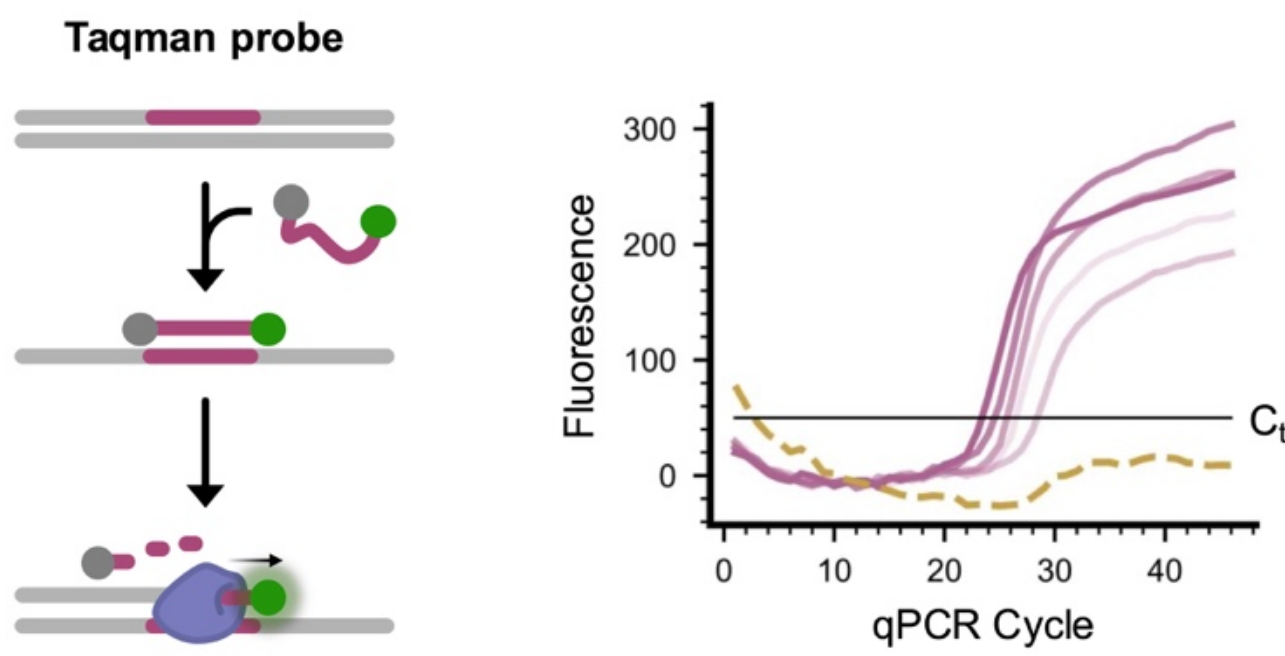

Supplementary Figure S11. Conversion of target RNA to DNA by squaramide ligation for mRNA site 2. Reactions were performed with target RNA (300, 30, 0.3, 0.03, 0.003 femtomoles) in the presence of 100 ng total RNA for 15 min at 50 °C using a TaqMan probe. Yellow dashed line is the ‘no template’ negative control. The oligonucleotides used for ligation against mRNA site 2 and qPCR as well as the target sequence can be found in Supplementary Table S3, while the sequence of the TaqMan probe is in Supplementary Table S5.

Supplementary Table S1. Oligonucleotides used to detect mRNA target Site 1 for the single ligation system. Oligonucleotide hybridisation to the target mRNA is colour coded (yellow and teal), while primer binding region on the squaramide forming oligonucleotides are underlined. Chemical modifications are shown at the end of the table.

Code	Sequence (5'-3')	Mol. Weight (g/mol)	
		Expected	Found
SL1-Am3	AGCAGCTGTACTAGATCTCGGCGTGTCTC**-3'-dT-NH$_2$**	9188	9189
SL1-Am3-SQ	AGCAGCTGTACTAGATCTCGGCGTGTCTC**-3'-dT-mono-sq**	9298	9300
SL1-Am5	**5'-dT-NH$_2$-**ATTGATGGCATCGGTCAGGGCTACTTAGC	9252	9253
SL1-Single-Primer F	AGCAGCTGTACTAGATCTCG	6117	6118
SL1-Single-Primer R	GCTAAGTAGCCCTGACCG	5485	5485

mRNA Target Sequence:

5'-....GGACAGACAA***CCUGACCGAUGCCAUCAAUAAGAGACACGCCGAGAUCUA***CAAGGGCCUGU....-3'

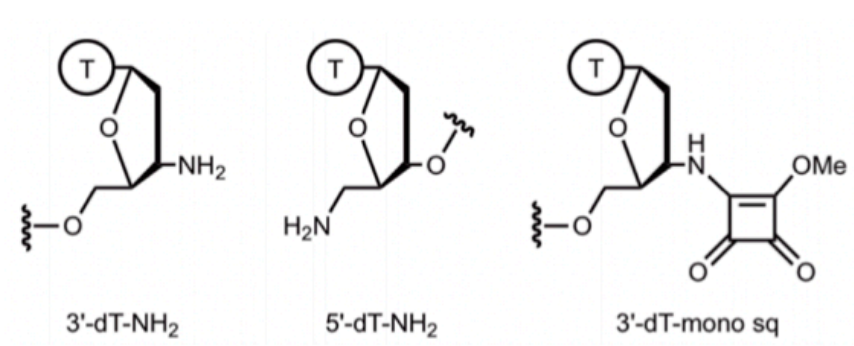

Supplementary Table S2. Oligonucleotides used to detect mRNA target Site 1 for the dual ligation system. Oligonucleotide hybridisation to the target mRNA is colour coded (teal, orange and red), while primer binding region on the squaramide forming oligonucleotides are underlined. For molecular beacons, stem formation regions are in black with the probe sequence that hybridises to the ligation product in orange. Chemical modifications are shown at the end of the table. Note that apart from optimisation, DL1-Molecular Beacon-AA was used for all qPCR reactions that require molecular beacons for this design.

Code	Sequence (5'-3')	Mol. Weight (g/mol)	
		Expected	Found
DL1-Am3 (res8375)	AGCAGCTGTACTAGATCTCGGCGTGTCTC**-3'-dT-**NH_2	9188	9189
DL1-Am5-Am3	**5'-dT-**NH_2**-**ATTGATGGCATCGGTCAGG**-3'-dT-**NH_2	6490	6491
DL1-Am5-Am3-SQ	**5'-dT-mono-sq-**ATTGATGGCATCGGTCAGG**-3'-dT-mono-sq**	6710	6712
DL1-Am5	**5'-dT-**NH_2**-**GTCTGTCCGGCCGATGAAGTGCTACTTAGC	9518	9519
DL1-Primer F	AGCAGCTGTACTAGATCTCG	6117	6118
DL1- Primer R	GCTAAGTAGCACTTCATCGG	6117	6118
DL1-Molecular Beacon-AA	**FAM-**AGCCGG ACCTGACCGATGCCATCAATACCGGCT**-Dabcyl**	11068	11069

mRNA Target Sequence:

5'-….GCCATCCACGACT ***ACTTCATCGGCCGGACAGACA ACCTGACCGATGCCATCAATAAGAGACACGCCGAGATCT***

ACAAGGGCCTGTTC….-3'

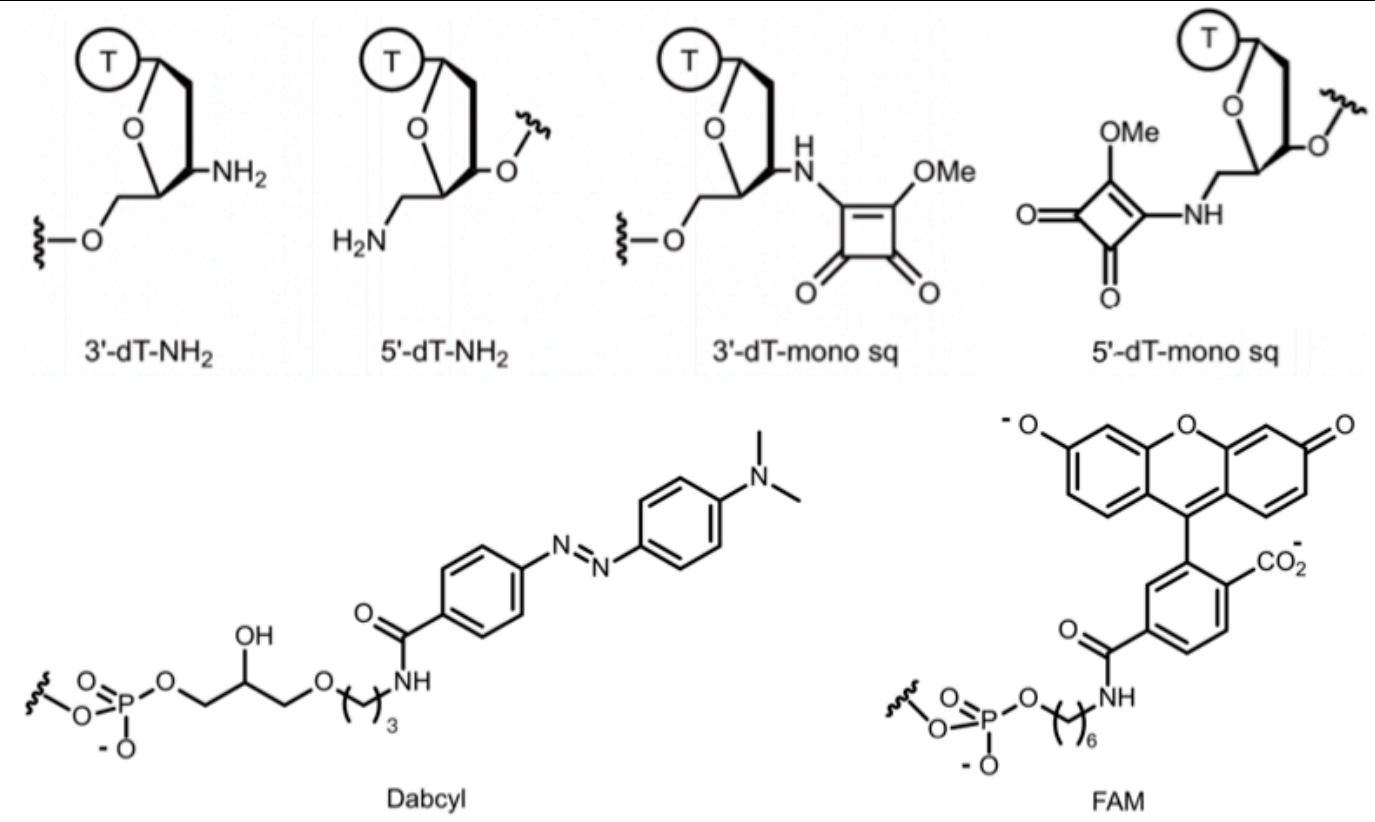
3'-dT-NH$_2$
5'-dT-NH$_2$
3'-dT-mono sq
5'-dT-mono sq
Dabcyl
FAM

Supplementary Table S3. Oligonucleotides used to detect mRNA target Site 2 for the dual ligation system. Oligonucleotide hybridisation to the target mRNA is colour coded (pink, purple and green), while primer binding region on the squaramide forming oligonucleotides are underlined. For molecular beacons, stem formation regions are in black with the probe sequence that hybridises to the ligation product in purple. Chemical modifications are shown at the end of the table. Note that apart from optimisation, DL2-Molecular Beacon-AA was used for all qPCR reactions that require molecular beacons for this design.

Code	Sequence (5'-3')	Mol. Weight (g/mol)	
		Expected	Found
DL2-Am3	GTCACTGGTCACAGATAGTACAGGCCG-**3'-dT-NH2**	8612	8613
DL2-Am5-Am3	**5'-dT-NH2-**CTTCACAAACAGGATGGCGCCA-**3'-dT-NH2**	7320	7320
DL2-Am5-Am3-SQ	**5'-dT-mono-sq-**CTTCACAAACAGGATGGCGCCA-**3'-dT-mono-sq**	7540	7541
DL2-Am5	**5'-dT-NH2-**GTTCTTCTCCTTATTCACGTCTGACCTACG	9353	9354
DL2-Primer F	GTCACTGGTCACAGATAGTAC	6430	6431
DL2-Primer R	CGTAGGTCAGACGTGAATAAG	6519	8520
DL2-Molecular Beacon-AA	**FAM-**GCCG ATGGCGCCATCCTGTTTGTGAAGA CATCGGC**-Dabcyl**	11764	11765

mRNA Target Sequence:

5'-.... ***ACGTGAATAAGGAGAAGAACA ATGGCGCCATCCTGTTTGTGAAGAACGGCCTGTACTATCTG***....-3'

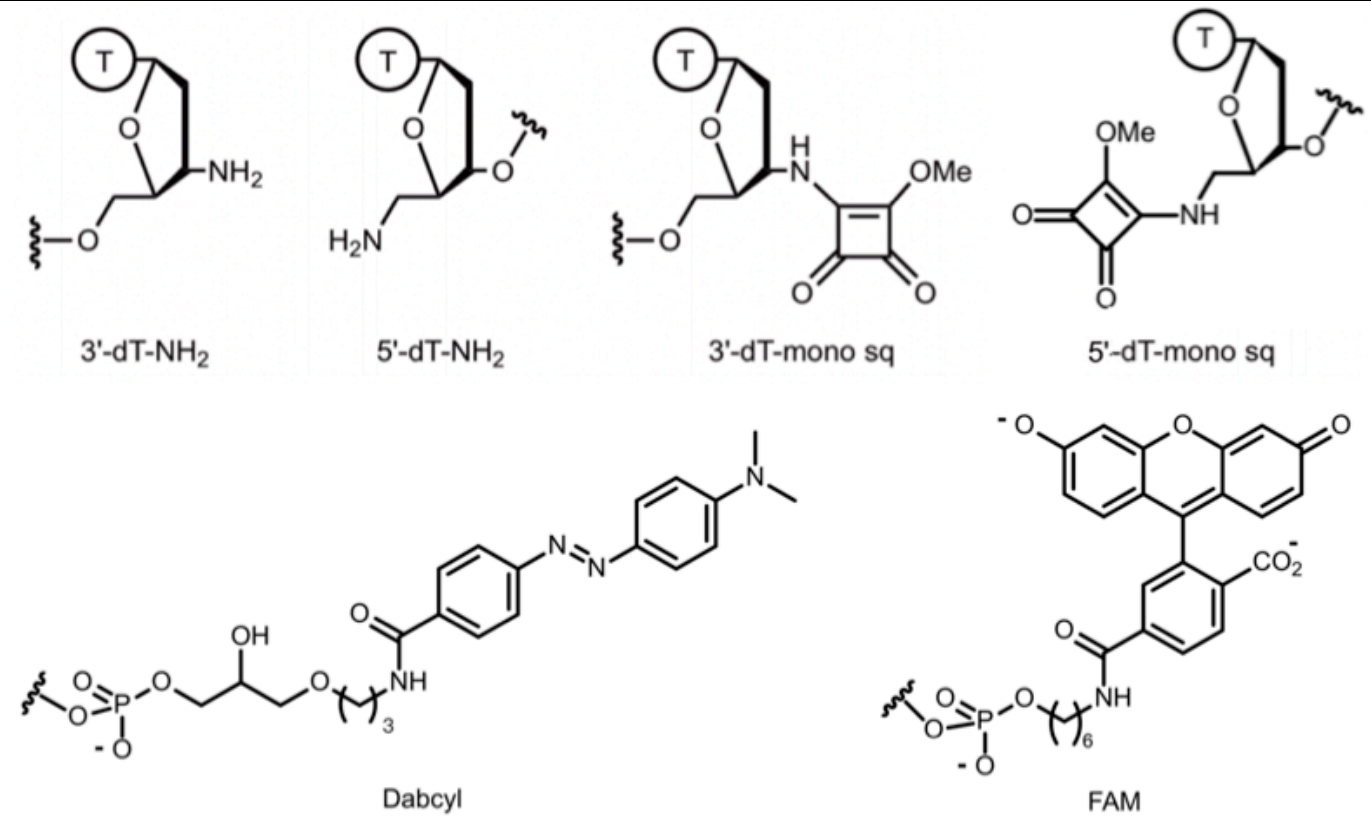
3'-dT-NH2
5'-dT-NH2
3'-dT-mono sq
5'-dT-mono sq
Dabcyl
FAM

Supplementary Table S4. Oligonucleotides used to directly evaluate squaramide ligation products on denaturing PAGE. DNA templates used to generate IVT RNA for squaramide ligation are colour coded (pink, purple and green) to highlight hybridisation positions of the ligation oligonucleotides. The black underlined region of the DNA templates corresponds to the T7 promoter sequence. Red highlighted bases are point mutations of the full complementary DNA Template 1.

Code	Sequence (5'-3')
DL3-Am3	TGGGGTACTGATGCAACAG**-3'-dT-NH_2**
DL3-Am5-Am3	**5'-dT-NH_2-** GGGTAGCCAATCTGCAGACAGACACTGGCAACA**-3'-dT-NH_2**
DL3-Am5	**5'-dT-NH_2-**GCGGACACCCTCCAGGAAGC
DNA Template 1 (Mismatch 0)	TTAATGTGATGGGGTACTGATGCAACAGTTGGGTAGCCAATCT<br>GCAGACAGACACTGGCAACATTGCGGACACCCTCCAGGAAGC<br>CCTATAGTGAGTCGTATTA
DNA Template 2 (Mismatch 1)	TTAATGTGATGGGGTACTGATG AACAGTTGGGTAGCCAATCT<br>GCAGACAGACACTGGCAACATTGCGGACACCCTCCAGGAAGC<br>CCTATAGTGAGTCGTATTA
DNA Template 3 (Mismatch 2)	TTAATGTGATGGGGTA TGATG AACAGTTGGGTAGCCAATCT<br>GCAGACAGACACTGGCAACATTGCGGACACCCTCCAGGAAGC<br>CCTATAGTGAGTCGTATTA
DNA Template 4 (Mismatch 3)	TTAATGTGATGG GTA TGATG AACAGTTGGGTAGCCAATCT<br>GCAGACAGACACTGGCAACATTGCGGACACCCTCCAGGAAGC<br>CCTATAGTGAGTCGTATTA
T7 Promoter	TAATACGACTCACTATAGG

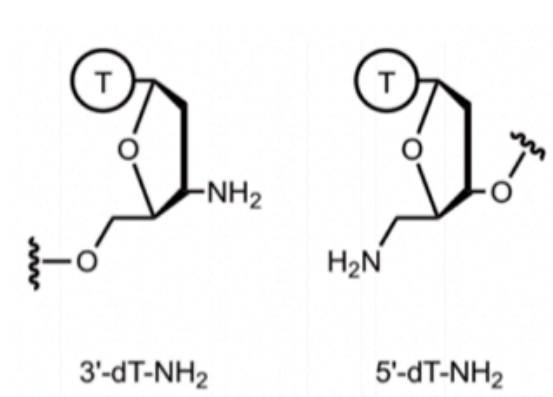

Supplementary Table S5. The TaqMan probe used to detect mRNA target Site 2 in the dual ligation. All sequences for ligation and amplification are identical to those in Supplementary Table S3 except instead of using the Molecular Beacon, the TaqMan probe used instead. The sequence that hybridises to the ligation product in purple. Chemical modifications are shown at the end of the table.

Code	Sequence (5'-3')	Mol. Weight (g/mol)	
		Expected	Found
DL2-TaqMan	**FAM-**GCGCCATCCTGTTTGTGAAGA**-Dabcyl**	7527	7528

mRNA Target Sequence:

5'**-….** ***ACGTGAATAAGGAGAAGAACA***

ATGGCGCCATCCTGTTTGTGAAGAACGGCCTGTACTATCTG….-3'

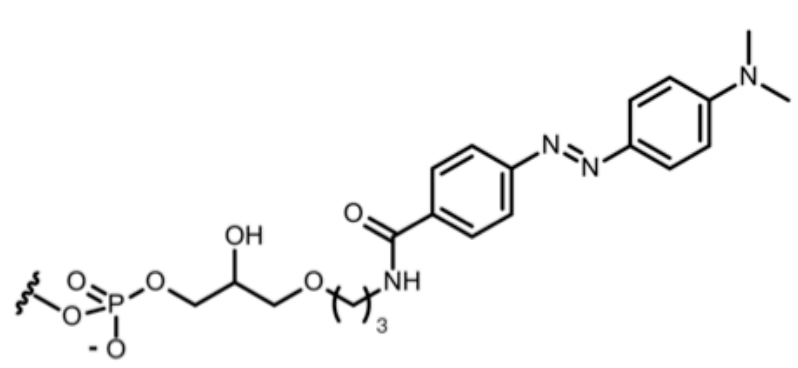

Dabcyl

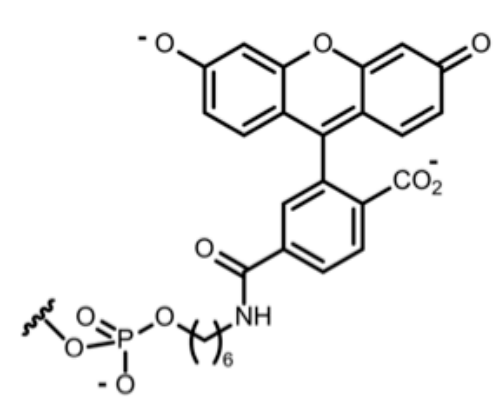

FAM
