# Supplementary material for: Non-enzymatic conversion of RNA sequence information into DNA by squaramide ligation for accurate RNA quantification
Source: Commun Chem. 2026 May 19;9:272. doi: 10.1038/s42004-026-02069-5 (PMC13454357; doi:10.1038/s42004-026-02069-5)
Supplement: Supplementary file 3 [file 42004_2026_2069_MOESM3_ESM.pdf]

Description of Additional Supplementary Files:

File name: Supplementary Data 1

Description: All numerical data for graphs presented in the manuscript and supplementary information.
